# The impact of prior level of care on the course of proximal humeral fractures in older patients: an analysis based on health insurance claims data

**DOI:** 10.1186/s12913-026-14024-0

**Published:** 2026-02-11

**Authors:** Janette Iking, J. Christoph Katthagen, Jeanette Koeppe, Karen Fischhuber, Jan P. Happe, Ursula Marschall, Michael J. Raschke, Josef Stolberg-Stolberg

**Affiliations:** 1 Department of Trauma, Hand and Reconstructive Surgery, University Hospital Münster, Albert-Schweitzer-Campus 1, Building W1, 48149 Münster, Germany; 2 Research Group “Mathematical Surgery”, University Hospital Münster, University of Münster, Münster, Germany; 3 Institute of Biostatistics and Clinical Research, University of Münster, Schmeddingstraße 56, 48149 Münster, Germany; 4 BARMER Institute for Health System Research, Lichtscheider Straße 89, 42285 Wuppertal, Germany

**Keywords:** proximal humeral fracture, level of care, Geriatric surgery, complication rates, Real-world analysis, Multivariable regression analysis, Health service research

## Abstract

**Background:**

The proximal humeral fracture (PHF) is the third most common fracture in older individuals. Prior level of care (LoC) and associated comorbidities may have an impact on patient outcome and prognosis.

**Methods:**

Retrospective German health insurance data from patients with PHF aged 65 years and older between 01/17 to 09/22 were analysed. The primary endpoints included overall survival (OS), major adverse events (MAEs), thromboembolic events (TEs), and surgery- or injury-related complications. All endpoints were analysed using multivariable models.

**Results:**

A total of 55,798 patients (median age 79 years; 84% female) were included. Prior to PHF, 68% had no LoC (LoC I 3%, LoC II 12%, LoC III 11%, LoC IV 6%, LoC V 1%), and 8% were living in a nursing home. With increasing LoC, the proportion of patients receiving non-operative treatment (no LoC 52%, LoC I 53%, LoC II 62%, LoC III 64%, LoC IV 71%, LoC V 76%) and the likelihood of a worse outcome increased. Both, mortality rates (1-year mortality: no LoC 4%, LoC I 12%, LoC II 19%, LoC III 29%, LoC IV 41%, LoC V 50%) and rates of MAEs increased drastically with increasing LoC. Multivariable analyses confirmed that increasing LoC was associated with a greater risk of death, MAEs, and TEs (all *p* < 0.001).

**Conclusion:**

Prior LoC has a significant effect on the course of PHF and the choice of treatment method in older individuals. This should be considered when making treatment decisions.

**Level of evidence:**

Level III, retrospective comparative study.

**Supplementary information:**

The online version contains supplementary material available at 10.1186/s12913-026-14024-0.

## Introduction

The proximal humeral fracture (PHF) is the third most common fracture in patients aged 65 years and older [1–6]. German health insurance data, including in- and outpatient data, report incidences of up to 350 per 100,000 person-years. As PHF is not only age- but also osteoporosis-associated, older women are even more affected, with incidences as high as 500 per 100,000 person-years [7]. These numbers are likely to increase due to demographic changes [6]. Interestingly, there is no consensus among surgeons regarding which treatment option is ideal for older patients with PHF [8]. Patient-individual factors such as comorbidities [9, 10], age [11], bone quality [12], male sex [10, 13], treatment method [14, 15], and previous level of care (LoC) [16] might be decisive for the outcome and prognosis after PHF.

The German statutory long-term care insurance (LTCI) system is based on five levels of care ranging from LoC I for those with minimal care needs to LoC V for patients with the most severe care requirements, who are often confined to a bed or wheelchair [17]. These levels determine the amount of financial support and services individuals receive to help them with daily activities and healthcare. In 1996, approximately 1.5 million people received support from the German LTCI. As of December 2022, approximately 4.9 million people in Germany received financial healthcare support [18], and the number is expected to rise along with an ageing population. Thus, LoC is not only an administrative classification but also provides a standardized measure of frailty and dependency within the German healthcare system. Compared to other international settings, LoC can be considered conceptually similar to functional status or frailty assessments used elsewhere, making it a valuable variable for stratifying outcomes in older patients.

Especially in the context of age-related fractures, however, little is known about the association between a pre-existing LoC and the course after fracture treatment [10, 16]. Detailed, stratified analyses regarding LoC in a large cohort of older people over a long period of time are lacking. Thus, the aim of this study was to analyze the influence of the LoC of older patients on the course of PHF. It was hypothesized that an increase in LoC leads to increased mortality, major adverse events (MAEs) and thromboembolic events (TEs). Additionally, we investigated the influence of LoC on minor outpatient complications and secondary osteoporosis-associated fractures.

## Materials and methods

### Data pool and patient cohort

The study was based on anonymized claims data from BARMER, one of the largest statutory health insurance providers in Germany, covering approximately 9 million individuals (roughly 11% of the German population). The dataset is therefore broadly representative of the general German population covered by statutory insurance. As described in previously published studies, the remuneration system in Germany is based on the “German Diagnosis Related Groups” (G-DRG) system, which is specified and regulated by mandatory coding instructions, including encoded diagnoses (International Statistical Classification of Diseases, German Modification; ICD-10 GM) and procedures (German operation and procedure classification; *Operationen- und Prozedurenschlüssel*, OPS) [2, 15, 19].

For this study, patient remuneration data (inpatient and outpatient data) of the BARMER health insurance from 2005 to 2022 were available. All older patients (aged ≥65 years) with in- or outpatient-coded PHF (ICD-10 code S42.2) between 01/2017 and 09/2022 were included (*n* = 55,798). The first coded diagnosis of PHF was defined as the index event of the study. Fracture complexity was approximated based on OPS subclassifications, differentiating simple (two-part) and multi-fragment (three- or four-part) fractures. Patients with prior PHF within five years before the index event were excluded. Further exclusion criteria were incomplete basic information or insurance status within 2 years before the index event, age < 65 years, coded polytrauma, and pathological fractures associated with bone tumors or metastases (see Additional file 1). With a median follow-up of 37.5 months, patients were observed from the date of fracture to the end of follow-up at the end of the study (31.12.2022), exit from the database, or death.

### Covariates

Demographic variables included age (as a continuous variable), sex, and the year of fracture. Fracture severity was not included as a covariate in the multivariable models. Although ICD-10 GM codes distinguish between fracture sites, they do not provide sufficient information to reliably determine displacement or comminution. Information regarding surgical treatment methods was obtained via the German OPS coding system, with special emphasis on locking plate fixation (LPF; 5–793.k1, 5–793.31, 5–794.21, 5–794.k1) and reverse total shoulder arthroplasty (RTSA; 5–824.21). Treatment allocation was considered by including surgical treatment within 21 days after PHF, which was modelled as a time-dependent covariate. Non-operative therapy was defined as the absence of surgical treatment within the first 21 days after the diagnosis of PHF. In addition to locking plate fixation (LPF) and reverse total shoulder arthroplasty (RTSA), we grouped all other surgical procedures under the category “other forms of surgery.” This included open reduction without internal fixation, intramedullary nailing, screw or cerclage fixation, and hemiarthroplasty, as defined by specific OPS codes (see Additional file 2). The comorbidity profile was determined using ICD-10 GM and OPS codes documented during the index hospitalization as well as in the 24 months prior to the fracture (in-patient and out-patient data; see Additional file 2). The hospital frailty score was determined on the basis of prior clinical diagnoses coded with the ICD-10 system, but without modification to the German ICD-10 version. A numerical score is therefore calculated by the number of relevant ICD-10 codes from a patient’s prior diagnosis. The hospital frailty score can be classified as low ( < 5 points), intermediate (5–15 points), or high ( > 15 points) [20]. In addition, pharmacotherapy prior to PHF was considered, including prescriptions of anticoagulants, vitamin D/calcium supplements, and bisphosphonates, which were identified using Anatomical Therapeutic Chemical Classification (ATC) codes (see Additional file 2). These covariates were chosen to adjust for differences in baseline characteristics, health status, and treatment allocation across LoC groups.

### Pre-existing LoC and the German LTCI system

Pre-existing LoC was detected within two years before PHF, using the latest reported level. In Germany, the statutory long-term care insurance (LTCI) system defines five levels of care (“Pflegegrade”), ranging from Level I (minor impairment) to Level V (most severe impairment). These levels are granted following a standardized medical assessment and reflect a patient’s ability to perform activities of daily living and the extent of support required:

- LoC I: Minor limitations in independence with occasional assistance needed.
- LoC II: Significant impairments requiring daily support.
- LoC III: Severe impairments requiring extensive assistance with most activities.
- LoC IV: Very severe impairments with patients often depending on comprehensive daily care, often living in nursing home care.
- LoC V: Most severe impairments with patients often bedbound or wheelchair-bound, requiring full-time nursing care, often living in nursing home care.

Assignment to a LoC determines the amount of financial support and care services covered by LTCI. For this study, information on pre-existing LoC was extracted from the claims data, which systematically records the LTCI level granted to each insured person.

### Primary endpoints

For the present study, overall survival (OS; defined by time to death from any cause), MAEs (defined as resuscitation, acute myocardial infarction, cardiac arrest, stroke, sepsis, acute renal failure, acute liver failure, acute respiratory distress syndrome, or death), TEs (defined as deep vein thrombosis, pulmonary embolism, ischaemic stroke, or death) and surgical or injury-related complications (for definition, see Additional file 2) were defined as the primary endpoints.

### Secondary endpoints

Minor outpatient complications and secondary osteoporosis-associated fractures were defined as secondary endpoints and are explained in more detail in Additional file 2.

### Missing values

Except for missing information about basic data, such as sex, date of birth, or date of death (which were defined as exclusion criteria), no data were missing in the study, since all variables were defined by existing ICD-10 or OPS codes. If no related code was found, the variable was set to zero.

### Statistical methods

Patients were grouped by their pre-existing LoC. For OS, MAEs, and TEs, survival functions were determined via Kaplan‒Meier estimates. The associations between LoC and OS/MAEs/TEs were modelled via multivariable Cox proportional hazards models, including LoC, sex, age, year of fracture, surgical treatment within 21 days after PHF (yes/no as a time-dependent variable) and patient comorbidity profile at PHF. Hazard ratios (HR) and 95% confidence intervals (95% CI) are presented.

For secondary osteoporosis-associated fractures, surgery- or injury-related complications, and minor outpatient complications, death was considered a competing risk event. Hence, cumulative incidence functions were determined by Aalen-Johansen estimates. The associations between LoC and secondary osteoporosis-associated fractures, surgery- or injury-related complications, and minor outpatient complications were modelled via multivariable Fine and Gray models, and subdistributional HRs with 95% CIs are presented. In addition, time trends in the associations between treatment and surgery- or injury-related complications were further modelled with a time-dependent treatment variable. The treatment variable depending on the time passed since PHF was evaluated via a step function.

To account for possible differences in the treatment effect on the outcomes between different LoCs, all analyses were repeated with an additional interaction term: surgical treatment*LoC. The full results of all the multivariable models are presented in Additional file 3. All multivariable models were adjusted for the presented set of covariates selected on the basis of clinical relevance and prior literature on outcomes after proximal humeral fractures.

All analyses were fully explorative without adjustment for multiple comparisons, and all p-values were interpreted accordingly in the sense of hypothesis generation. Statistical analyses were performed using SAS Enterprise Guide Version 8.3 Update 2 (SAS Institute Inc., Cary, NC, USA) and R version 4.2.0 (2022–04–22, R Foundation for Statistical Computing, Vienna, Austria).

## Results

### Baseline characteristics & comorbidities

In total, 55,798 patients (83.7% female, median age 79 years) were included in the study. Prior to PHF, 37,906 (67.9%) had no LoC, 1387 (2.5%) had LoC I, 6569 (11.8%) had LoC II, 5907 (10.6%) had LoC III, 3232 (5.8%) had LoC IV, and 797 (1.4%) had LoC V. 8% (*n* = 4,451) of the entire cohort already lived in a nursing home prior to PHF. Patients without a LoC were equally represented in the inpatient and outpatient sectors (in: 19,617 (51.8%) vs. out: 18,289 (48.3%)), whereas patients with a LoC were more likely to be treated in the inpatient sector (in: 12,258 (68.5%) vs. out: 5634 (31.5%)). In total, 31,256 (56.0%) patients were treated non-operatively. The most common comorbidities in the cohort were hypertension (46,174 (82.8%)), diabetes mellitus (16,953 (30.4%)), and cancer (16,003 (28.7%)). Most comorbidities increased in frequency with increasing LoC (see Table 1).

**Table 1:** Patient characteristics at diagnosis

	Entire Cohort	No LoC	LoC I	LoC II	LoC III	LoC IV	LoC V
Frequency – n (%)	55,798 (100.0%)	37,906 (67.9%)	1,387 (2.5%)	6,569 (11.8%)	5,907 (10.6%)	3,232(5.8%)	797(1.4%)
*Sector of first diagnosis–n(%)*							
Inpatient sector	31,875 (57.1%)	19,617 (51.8%)	936 (67.5%)	4,430 (67.4%)	4,178 (70.7%)	2,199 (68.0%)	515 (64.6%)
Outpatient sector	23,923 (42.9%)	18,289 (48.3%)	451 (32.5%)	2,139 (32.6%)	1,729 (29.3%)	1,033 (32.0%)	282 (35.4%)
Non-operative treatment–n(%)	31,256 (56.0%)	19,819 (52.3%)	740 (53.4%)	4,037 (61.5%)	3,774 (63.9%)	2,283 (70.6%)	603(75.7%)
*Surgical treatment within 21 days after PHF – n (% total)*							
Any	24,542 (44.0%)	18,087 (47.7%)	647 (46.7%)	2,532 (38.5%)	2,133 (36.1%)	949 (29.4%)	194 (24.3%)
sLPF	1,755 (3.2%)	1,302 (3.4%)	46 (3.3%)	168 (2.6%)	140 (2.4%)	85 (2.6%)	14 (1.8%)
LPF	11,233 (20.1%)	8,779 (23.2%)	248 (17.9%)	986 (15.0%)	812 (13.8%)	349 (10.8%)	59 (7.4%)
RTSA	7,122 (12.8%)	5,011 (13.2%)	246 (17.7%)	892 (13.6%)	687 (11.6%)	232 (7.2%)	54 (6.8%)
Other	8,504 (15.2%)	6,137 (16.2%)	191 (13.8%)	879(13.4%)	799 (13.5%)	411 (12.7%)	87 (10.9%)
*Surgical treatment within 21 days after PHF – n (% OP)*							
Any	24,542 (100.0%)	18,087 (100.0%)	647 (100.0%)	2,532 (100.0%)	2,133 (100.0%)	949 (100.0%)	194 (100.0%)
sLPF	1,755 (7.2%)	1,302 (7.2%)	46 (7.1%)	168 (6.6%)	140 (6.6%)	85 (9.0%)	14 (7.2%)
LPF	11,233 (45.8%)	8,779 (48.5%)	248 (38.3%)	986 (38.9%)	812 (38.1%)	349 (36.8%)	59 (30.4%)
RTSA	7,122 (29.0%)	5,011 (27.7%)	246 (38.0%)	892 (35.2%)	687 (32.2%)	232 (24.5%)	54 (27.8%)
Other	8,504 (34.7%)	6,137 (33.9%)	191 (29.5%)	879 (34.7%)	799 (37.5%)	411 (43.3%)	87 (44.9%)
Nursing home before PHF – *n* (%)	4,451 (8.0%)	6 (0.0%)	21 (1.5%)	744 (11.3%)	1,725 (29.2%)	1,522 (47.0%)	433 (54.3%)
Median residence time in nursing home before PHF – month (Q1, Q3)	20.5 (7.3, 42.9)	20.5 (6.8, 60.9)	15.0 (5.4, 26.4)	16.9 (5.8, 35.0)	17.7 (6.3, 38.3)	22.6 (8.6, 45.8)	34.2 (16.0, 61.8)
Median Hospital Frailty Risk score (Q1, Q3)	9.9 (4.5, 18.8)	6.7 (3.2, 11.9)	15.9 (9.6, 22.7)	18.2 (11.4, 26.2)	23.4 (15.6, 32.7)	25.5 (19.35, 37.8)	29.1 (20.7, 39.8)
Median age – years (Q1, Q3)	79 (73, 85)	77 (71, 82)	82 (77, 86)	84 (79, 89)	85 (80, 90)	86 (81, 91)	86 (80, 91)
Age ≥ 80 years – *n* (%)	27,235 (48.8%)	13,752 (36.3%)	905 (65.3%)	4,801 (73.1%)	4,574 (77.4%)	2,592 (80.2%)	611 (76.7%)
Female sex – *n* (%)	46,694 (83.7%)	31,950 (84.3%)	1,184 (85.4%)	5,481 (83.4%)	4,799 (81.2%)	2,619 (81.0%)	661(82.9%)
Osteoporosis – *n* (%)	19,580 (35.1%)	11,671 (20.9%)	652 (47.0%)	3,007 (45.8%)	2,635 (44.6%)	1,318 (40.8%)	297 (37.3%)
*No of prior osteoporosis-assoc. fractures within 5 years before PHF – n (%)*							
0	42,658 (76.5%)	31,749 (83.8%)	910 (65.6%)	4,145 (63.1%)	3,546 (60.0%)	1,833 (56.7%)	475 (59.6%)
1	9,932 (17.8%)	5,016 (13.2%)	338 (24.4%)	1,713 (26.1%)	1,638 (27.7%)	995 (30.8%)	232 (29.1%)
2	2,543 (4.6%)	979 (2.6%)	112 (8.1%)	544 (8.3%)	543 (9.2%)	303 (9.4%)	62 (7.8%)
3	567 (1.0%)	146 (0.4%)	21 (1.5%)	149 (2.3%)	145 (2.5%)	83 (2.6%)	23 (2.9%)
4	96 (0.2%)	16 (0.0%)	6 (0.4%)	16 (0.2%)	35 (0.6%)	18 (0.6%)	5 (0.6%)
*Type of prior osteoporosis-assoc. fractures within 5 years before PHF – n (%)*							
Distal radius	1,079 (1.9%)	612 (1.6%)	36 (2.6%)	154 (2.3%)	167 (2.8%)	87 (2.7%)	23 (2.9%)
Proximal femur	6,005 (10.8%)	2,242 (5.9%)	197 (14.2%)	1,188 (18.1%)	1,291 (21.9%)	874 (27.0%)	213 (26.7%)
Vertebrae	6,302 (11.3%)	3,049 (8.0%)	259 (18.7%)	1,226 (18.7%)	1,090 (18.5%)	556 (17.2%)	122 (15.3%)
Pelvic ring fractures	2,476 (4.4%)	1,060 (2.8%)	101 (7.3%)	469 (7.1%)	512 (8.7%)	275 (8.5%)	59 (7.4%)
*Further comorbidities:*							
Cancer – *n* (%)	16,003 (28.7%)	10,514 (27.7%)	454 (32.7%)	2,128 (32.4%)	1,814 (30.7%)	892 (27.6%)	201 (25.2%)
Diabetes mellitus – *n* (%)	16,953 (30.4%)	10,305 (27.2%)	522 (37.6%)	2,547 (38.8%)	2,139 (36.2%)	1,158 (35.8%)	282 (35.4%)
Dementia – *n* (%)	7,553 (13.5%)	1,368 (3.6%)	152 (11.0%)	1,186 (18.0%)	2,329 (39.4%)	2,000 (61.9%)	518 (65.0%)
Chronic polyarthritis – *n* (%)	3,683 (6.6%)	2,416 (6.4%)	126 (9.0%)	534 (8.1%)	422 (7.1%)	150 (4.6%)	35 (4.4%)
Obesity – *n* (%)	11,782 (21.1%)	8,177 (21.6%)	363 (26.2%)	1,616 (24.6%)	1,074 (18.2%)	444(13.7%)	108 (13.6%)
Nicotin abuse – *n* (%)	4,299 (7.7%)	3,011 (7.9%)	141 (10.2%)	524 (8.0%)	411 (7.0%)	179 (5.5%)	33 (4.1%)
Parkinson – *n* (%)	2,156 (3.9%)	562 (1.5%)	54 (3.9%)	448 (6.8%)	579 (9.80%)	404 (12.5%)	109 (13.7%)
Rotator cuff rupture – *n* (%)	*** (2.6%)	1,179 (3.1%)	38 (2.7%)	141 (2.2%)	77 (1.3%)	33 (1.0%)	<5(***)
Alcohol abuses – *n* (%)	2,832 (5.1%)	1,763 (4.7%)	101 (7.3%)	404 (6.2%)	365 (6.2%)	168 (5.2%)	31 (3.9%)
Omarthrosis – *n* (%)	1,890 (3.4%)	1,229 (3.2%)	53 (3.8%)	243 (3.7%)	236 (4.0%)	106 (3.3%)	23 (2.9%)
Frozen shoulder – *n* (%)	2,194 (3.9%)	1,609 (4.2%)	62 (4.5%)	261 (4.0%)	171 (2.9%)	78 (2.4%)	13 (1.6%)
Atrial fibrillation/flutter – *n* (%)	11,456 (20.5%)	5,696 (15.0%)	406 (29.3%)	2,138 (32.6%)	1,926 (32.6%)	1,060 (32.8%)	230 (28.9%)
Congestive heart failure – *n* (%)	13,592 (24.4%)	6,310 (16.7%)	501 (36.1%)	2,643 (40.2%)	2,494 (42.2%)	1,336 (41.3%)	308 (38.6%)
Coronary heart disease – *n* (%)	13,762 (24.7%)	7,668 (20.2%)	455 (32.8%)	2,323 (35.4%)	2,061 (34.9%)	1,029 (31.8%)	226 (28.4%)
Hypertension – *n* (%)	46,174 (82.8%)	30,020 (79.2%)	1,241 (89.5%)	6,000 (91.4%)	5,378 (91.0%)	2,852 (88.2%)	683 (85.7%)
Atherosclerosis – *n* (%)	9,966 (17.9%)	6,028 (15.9%)	307 (22.1%)	1,560 (23.8%)	1,323 (22.4%)	629 (19.5%)	119 (14.9%)
Stroke and/or CVD before PHF – *n* (%)	15,560 (27.9%)	8,200 (21.6%)	483 (34.8%)	2,449 (37.3%)	2,582 (43.7%)	1,477 (45.7%)	369 (46.3%)
Chronic kidney disease – *n* (%)	15,355 (27.5%)	7,566 (20.0%)	553 (39.9%)	2,856 (43.5%)	2,677 (45.3%)	1,390 (43.0%)	313 (39.3%)
*Previous medication:*							
Any anticoagulant – *n* (%)	18,830 (33.8%)	9,642 (25.4%)	644 (46.4%)	3,347 (51.0%)	3,140 (53.2%)	1,678 (51.9%)	379 (47.6%)
Vitamin D or Calcium – *n* (%)	4,893 (8.8%)	2,574 (6.8%)	190 (13.7%)	908 (13.8%)	791 (13.4%)	351 (10.9%)	79 (9.9%)
Bisphosphonates – *n* (%)	3,368 (6.0%)	2,073 (5.5%)	130 (9.4%)	516 (7.9%)	447 (7.6%)	176 (5.5%)	26 (3.3%)

Patients were classified based on pre-existing level of care (LoC). Proximal humeral fracture – PHF, locked plate fixation for multi-fragmented fracture – LPF, reverse total shoulder arthroplasty – RTSA, simple-fracture locked plate fixation – sLPF, CVD – cerebrovascular disease. *** censored due to data privacy protection

### Overall mortality and major adverse events

**Fig. 1:**
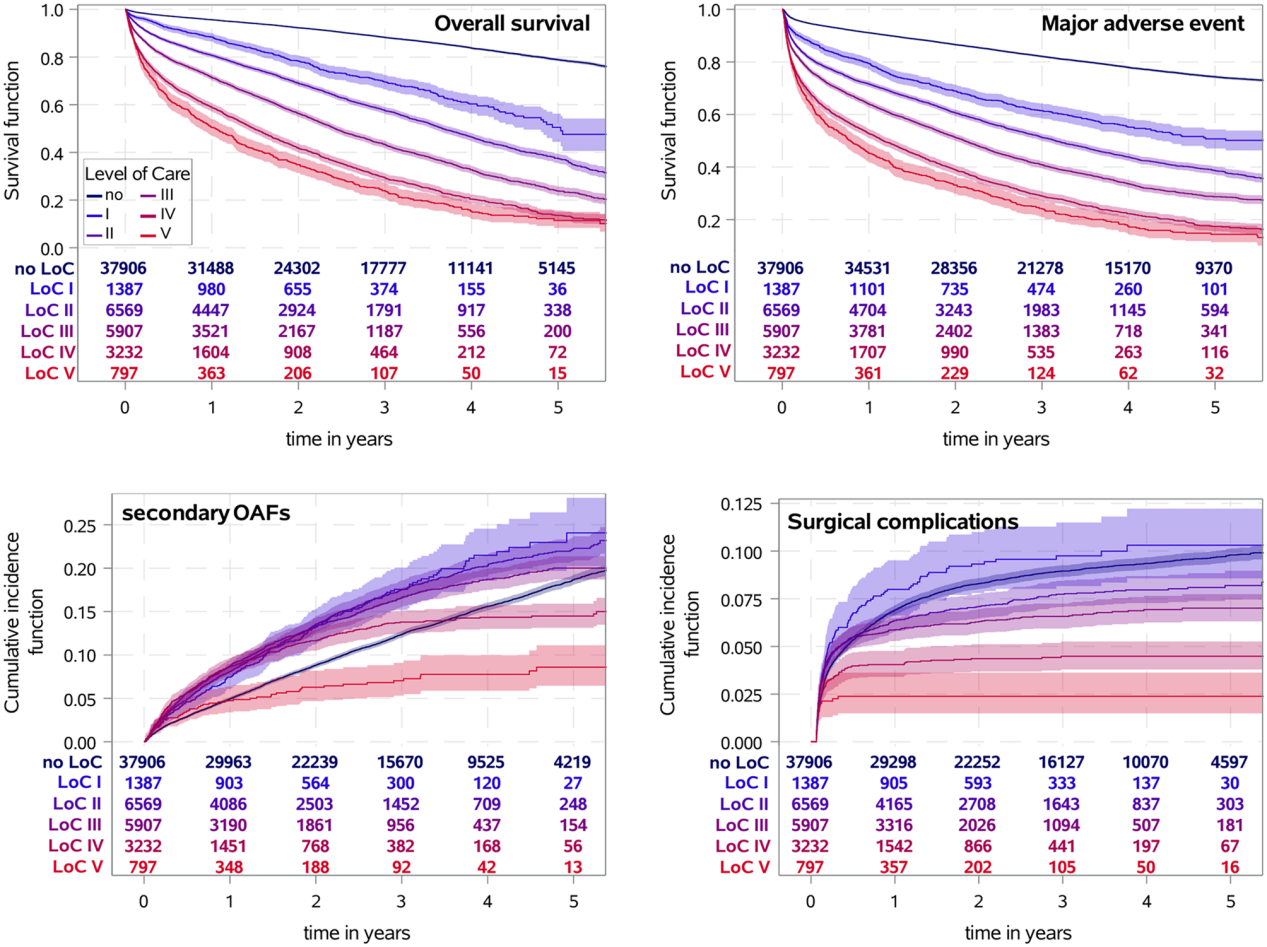
Event rates for primary endpoints after PHF depending on pre-existing level of care (LoC). For overall survival and major adverse events, the survival function (with 95% confidence interval) was determined using Kaplan-Meier estimate. For surgical complications and secondary osteoporosis-associated fractures (OAFs), death was considered a competing risk event, and the event rates were given by cumulative incidence functions (with 95% confidence intervals) determined using Aalen-Johansen estimates

**Fig. 2:**
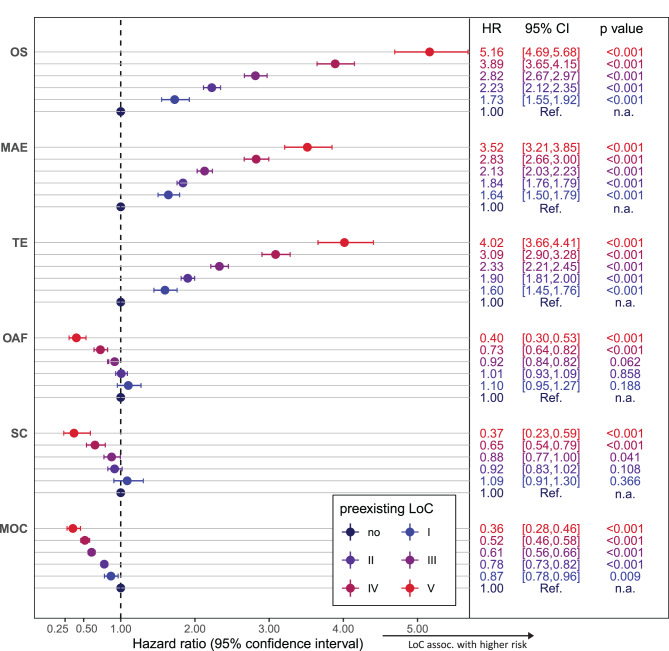
Multivariable analyses for primary and secondary endpoints. Modelling was performed using multivariable Cox proportional hazards regression analysis for overall survival (OS), major adverse events (MAEs), thromboembolic events (TEs) or death. In the case of all other endpoints, death was considered a competing risk event, and sub-distributional hazard ratios were determined using Fine and Gray models. Hazard ratios (HR) with 95% confidence intervals (95%CI) comparing LoC with reference to no pre-existing LoC are presented. Full results of the models are presented in Additional file 3

The likelihood of a worse outcome after PHF increased with increasing LoC (see Fig. 1, Additional file 4 and 5). At all times, the overall mortality increased drastically with increasing LoC (1^st^ year: no LoC: 4.4%; LoC I: 11.7%; LoC II: 19.3%; LoC III: 28.6%; LoC IV: 41.2%; LoC V: 49.6%). Similar associations were observed for MAEs and TEs. The descriptive results were also confirmed by multivariable analyses. Increasing LoC was associated with shorter OS, more MAEs, and more TEs during follow-up (all *p* < 0.001, Fig. 2). Moreover, for patients who underwent surgical treatment within 21 days after PHF, longer OS, fewer MAEs and TEs were observed (see Additional file 3), whereas no differences in the associations between specific treatments and outcomes were found for different LoCs (p_int_ > 0.05; see Additional file 5).

### Surgical and injury-related complications

In total, 24,542 (44.0%) patients were surgically treated, of whom 1755 (7.2%) received an LPF after a simple two-part fracture (sLPF), 11,233 (45.8%) received an LPF after a multi-fragment fracture, 7122 (29.0%) received an RTSA, and 8504 (34.7%) received another form of surgical treatment. With increasing LoC, the proportion of patients who underwent non-operative treatment increased (see Table 1). LPF was used in the majority of patients who were treated surgically in the lower LoC groups, whereas in the higher LoC groups, other forms of surgical treatment dominated. With increasing LoC, the use of other forms of surgical treatment increased (LoC I-III 35.2% vs. LoC IV-V 43.6%), and the use of RTSA decreased (LoC I-III 34.4% vs. LoC IV-V 25.0%), whereas the use of LPF remained relatively constant (LoC I-III 38.5% vs. LoC IV-V 35.7%). Surgery- or injury-related complications were observed in 6.5% of the individuals within one year after PHF (see Fig. 1, Additional file 4). The proportion of patients who experienced surgery- or injury-related complications decreased with increasing LoC. A similar pattern was observed when evaluating minor outpatient complications, which 24% of the whole cohort experienced 1 year after PHF. Multivariable analyses confirmed the descriptive results. With increasing LoC, fewer surgery- or injury-related complications (*p* < 0.001 for LoC IV and V) and minor outpatient complications (*p* < 0.001 for LoC II-V) were observed. Compared with patients without pre-existing LoC, individuals with the highest LoC (V) had the lowest rates of surgery- or injury-related complications (HR 0.37; 95% CI [0.23, 0.59]) and minor outpatient complications (HR 0.36; 95% CI [0.28, 0.46]; see Fig. 2). No differences were detected in the associations between specific treatments and surgery- or injury-related complications for different LoCs (p_int_ > 0.05), whereas surgically treated patients presented a reduced hazard ratio for minor outpatient complications with increasing LoC (p_int_ < 0.001, Additional file 5). Furthermore, stratified analyses of surgical complications revealed a time-dependent decrease in the risk for secondary interventions, with no significant differences between different LoCs (see Additional file 6 and 7).

### Prior and secondary osteoporosis-associated fractures

Patients with a prior LoC more often had a pre-existing diagnosis of osteoporosis (no LoC 20.9%, LoC ≥ I 44.2%), with a decreasing trend with increasing LoC (see Table 1). In accordance with that, individuals with a prior LoC also had a higher frequency of a prior osteoporosis-associated fracture (no LoC 13.2%; LoC ≥ I 27.5%). Fractures of the proximal femur (10.8%) and vertebrae (11.3%) were the most common prior osteoporosis-associated fractures in the cohort. The proportion of patients with a secondary osteoporosis-associated fracture within 5 years after PHF was greater in individuals with prior low LoC (no LoC 18.7%, LoC I 24.0%). When the ICD code M80 (osteoporosis with pathological fracture) was included, the proportion was almost twice as high (no LoC 28.8%, LoC I 40.1%; see Fig. 1, Additional file 4). The frequency of secondary osteoporosis-associated fractures tended to decrease with increasing LoC. The descriptive results were again confirmed by multivariable analyses. With increasing LoC, lower rates of secondary osteoporosis-associated fractures were observed (*p* < 0.001 for LoC IV and V compared to no LoC). Compared with patients without a pre-existing LoC, individuals with the highest LoC (LoC V) had the lowest rates of secondary osteoporosis-associated fractures (HR 0.40, 95% CI [0.30, 0.53]); see Fig. 2).

## Discussion

In this large nationwide cohort study, we found that higher LoC prior to fracture was strongly associated with adverse outcomes after PHF. Patients with higher LoC experienced increased mortality, major adverse events, and thromboembolic events, while the likelihood of surgical treatment declined markedly with increasing dependency. Within the surgical subgroup, RTSA and LPF were the predominant procedures, but the use of RTSA decreased with higher LoC. These findings suggest that LoC, as an administrative but clinically meaningful proxy for frailty and comorbidity burden, can be an important factor of treatment allocation and outcomes in older patients. Understanding this relationship underscores the need for individualized, risk-adapted decision-making in the management of proximal humeral fractures.

The PHF is one of the most common osteoporosis-associated fractures in older patients, however, the reported incidence rates of PHF vary greatly, depending, among other factors, on the age and sex distribution of the population as well as the location and time of counting [3, 4, 21]. In particular, data on patients who are treated non-operatively or have been diagnosed in the outpatient sector are often neglected. Recently, Koeppe et al. published detailed age- and sex-adjusted incidences of 351.1 (±7.7) PHF per 100,000 person-years, including all patients independent of treatment modality and patient sector [7]. This newly reported incidence is almost five times as high as some previously reported data, highlighting the importance of the outpatient sector and the societal and economic burden [5, 6]. In line, the majority of patients in this study were treated non-operatively (56%), and 43% of the patients were first diagnosed in the outpatient sector. With increasing LoC, more patients are treated in the inpatient sector, possibly because patients with a LoC already suffer from more functional limitations and comorbidities than patients without a LoC and therefore need immediate inpatient care. Other reasons for hospital admission may include the type and severity of the fracture, which might be affected by advanced age and increasing effects of osteoporosis, the need for surgical treatment, other concomitant injuries and comorbidities that require inpatient care, and the need for analgesic treatment [22].

The overall proportion of surgically treated patients in our cohort was higher than what has been reported in the United States [23, 24]. For example, a recent Medicare analysis found that only 6.5% of PHFs were managed operatively [25], while a broader nationwide geriatric cohort reported an operative rate of 37.6% [26]. In contrast, our German dataset showed that 44% of patients underwent surgical treatment within 21 days after fracture. Several factors may explain this discrepancy. German registry studies have consistently documented higher operative frequencies compared with the US [6, 15]. Possible reasons include differences in clinical practice patterns, surgeon training, guideline recommendations, and financial incentives inherent to the German DRG-based reimbursement system. Furthermore, the inclusion of both inpatient and outpatient cases in our dataset may contribute to the higher observed rates compared with US datasets, which often focus primarily on hospital-based cohorts. These structural and cultural differences between health systems may explain the higher operative frequencies observed in Germany compared with the US. Comparable differences can also be observed in other international settings. In Australia, approximately 30% of PHFs are treated surgically, while registry data from Sweden and Denmark report even lower operative rates of around 14% and 13%, respectively [27–29]. These findings underline that Germany’s higher surgical frequencies are not only distinct from the United States but also from other Western healthcare systems. Such variation most likely reflects differences in treatment culture, guideline recommendations, and health system incentives rather than patient characteristics alone.

Although the overall proportion of surgically treated patients in our German cohort was high compared with data from other countries, we observed a marked decline in operative treatment with increasing LoC, reaching up to 75% non-operative management in the highest LoC group. This trend aligns with the experience of many surgeons, who report that frail and multimorbid patients are more often managed non-operatively due to higher perioperative risks, limited functional reserve, and reduced rehabilitation potential. Non-operative treatment may also result from shared decision-making processes that incorporate patient preferences, surgeon experience, and perceived quality of life. At the same time, one must recognize that fracture characteristics are an important determinant of treatment choice: non-displaced PHFs are typically managed non-operatively, whereas displaced or multi-fragment fractures are often treated surgically [30]. However, as ICD-10 coding does not allow for reliable fracture classification, we cannot determine whether fracture complexity differed systematically across LoC groups. Moreover, given that older female patients are more likely to present with complex or displaced fractures [31], it seems unlikely that higher LoC groups suffered from less severe fracture types. Consequently, the high proportion of non-operative treatment in patients with prior LoC may also reflect a potential care deficit in this particularly vulnerable population.

The majority of surgically treated patients received LPF, which is consistent with previously reported studies [2, 16, 30], although the total number of PHFs treated with RTSA is constantly increasing [32–34]. Interestingly, we observed that the use of RTSA decreased with increasing LoC, which appears counterintuitive given that RTSA is often considered a preferred option in older and frailer patients with complex PHFs due to poor bone quality and reduced potential for stable osteosynthesis [35]. Several factors may explain this finding. First, surgeons may be reluctant to perform RTSA in patients with very high LoC because of their limited life expectancy, higher perioperative risk, and the greater rehabilitation requirements associated with prosthetic surgery. Second, “soft factors” such as overall frailty, comorbidity burden, functional goals, and anticipated compliance with postoperative care likely play an important role in surgical decision-making. Third, resource considerations, including the availability of specialized centres and surgical expertise, may further influence the observed pattern. As our dataset does not provide information on fracture morphology or surgeon preference, these explanations remain speculative, but they underline that treatment allocation in highly dependent patients is complex and cannot be fully captured by administrative data.

To date, there is no clear evidence as to which treatment option for PHF in older patients is superior, with only a small number of high-quality studies available [8]. In line with that, we did not observe any differences in the associations between the treatment method (surgical vs. non-operatively) and outcomes for different LoCs, indicating that surgery is not a risk factor for worse outcomes for a specific LoC. In contrast to Hammes et al. surgical treatment within 21 days after PHF was associated with better OS and less MAEs and TEs, suggesting that if surgery is needed, it should be scheduled in a timely manner [16]. Comparing the functional outcomes between surgically and non-operatively treated PHFs, neither recent systematic reviews nor high-quality randomized controlled trials, such as the ProFHER trial, have shown a significant difference, not even for displaced fractures [8, 36–38]. Although we cannot derive the functional outcome from our data, it has been shown that social independence is a predictive factor for functionality after PHF [39]. Patients who are not living in their own home, participate in recreational activities, are able to perform their own shopping, or are able to dress themselves have a significantly increased risk of a poor functional outcome (measured by the Constant–Murley score), which is independent of the severity of the fracture [39].

The 1-year mortality rate of the entire cohort was 11.6%, which is comparable to previously reported data of 12% from a large register study from Sweden for the same age group [28], higher than the mortality reported by Clement et al. (9.6% [39]), and Myeroff et al. (7.8% [11]), on the basis of much smaller cohorts, and lower than the reported 1-year mortality of Adam et al. (16.0% [10]). With increasing LoC, the likelihood of a worse outcome after PHF increased drastically, as mortality, MAE, and TE increased over time. A previous study from Hammes et al. analysing a cohort of 17,322 patients with PHF of all ages in the state of North Rhine-Westphalia (Germany) also reported that patients with prior LoC had increased 1-year mortality after PHF compared with patients without prior LoC; however, detailed, stratified analyses regarding LoC were lacking [16]. A study by Myeroff et al. revealed that unassisted ambulatory status (defined as unassisted, cane-assisted, walker-assisted, or wheelchair-confined) and living independence were both significant predictors of decreased mortality in older people with PHF. Patients relying on a cane or walker for mobility at baseline had the highest proportion of decreased ambulatory status after suffering from a PHF [11]. Similarly, another study showed that living at home is an independent predictor of survival in older patients with PHF [39].

A reason for the increased mortality in PHF patients with prior LoC could be a higher frequency of multiple long-term conditions in patients with LoC, which are associated with increased mortality [10, 11, 40]. Cancer, chronic obstructive pulmonary disease, or congestive heart failure can have an amplifying effect on mortality after PHF, as can infections and pneumonia [16]. Frailty, which is also associated with increased mortality, could be another factor that is more prevalent in older, multimorbid populations and increased in our study with increasing LoC [41]. What remains unclear, however, is whether patients with a prior LoC have a worse outcome because they are mainly treated non-operatively (which is associated with a worse outcome [10, 15]), or if non-operative treatment is associated with a worse outcome because, as a “rule of thumb” frail, multimorbid patients with low demand and poor health are often treated non-operatively [15, 42].

In contrast to mortality, the risk of major adverse events, thromboembolic events, surgery- or injury-related complications, minor outpatient complications, and secondary osteoporosis-associated fractures decreased with increasing LoC in this study. Thus, patients with a high pre-existing LoC had fewer secondary surgical treatments later in the disease course (including conversion from non-operative treatment to surgical fracture fixation or repeated surgery on the shoulder) after PHF. Moreover, these patients are less often treated in the outpatient sector because of minor complications. Both were also visible after adjustment for the patient’s risk profile and primary treatment decision. The reason for the reduced rates cannot be fully explained by the data. However, it is most likely explained by “soft” factors that drive decisions against (further) surgical treatment in this impaired and frail patient group. The lower rates of minor outpatient complications in patients with high LoC are more likely to indicate a care deficit than a better outcome and should be investigated in further studies.

Furthermore, the reduced risk for secondary osteoporosis-associated fractures in the LoC IV and V groups may be caused by the fact that, by definition, independence and mobility are severely limited in these patients. Thus, the risk of falling and therefore the risk for further fractures is decreased. Moreover, with a 40–50% 1-year mortality rate in patients with LoC IV and higher, these patients are much more likely to die before suffering from another fracture.

### Strengths and limitations

By analysing health claims data, it was possible to acquire one of the largest study populations on PHF at a German-wide level. Long-term observations, such as LoC, mortality, and complication rates, could be addressed. Further strengths of the study were the long-term follow-up; the use of a representative population cross-section; and the completeness of the data, including age, sex, and course. However, health claims data analysis also has certain limitations. For example, data were initially collected for financial purposes, not for scientific research. Hence, up-coding might influence the calculations and outcomes of this study, although this effect is likely to be the same across all groups. Furthermore, there might be a discrepancy between the prescribed medicine and the compliance of the patients to actually take it. As mentioned before, fracture classification and actual reasoning for treatment decisions cannot be determined from our data. The ICD-10 GM coding system does not differentiate between the degree of displacement or comminution of proximal humeral fractures with sufficient precision. While proxy indicators might be inferred from procedure codes, these approaches are prone to misclassification and were therefore not applied in our study. Consequently, potential confounding by fracture severity cannot be completely excluded. Furthermore, the LoC variable, although administratively well-defined within the German LTCI system, should be interpreted primarily as a proxy for patient frailty and comorbidity burden. Higher LoC levels are strongly associated with multimorbidity, functional dependency, and reduced independence. Consequently, our findings reflect associations between overall health status and outcomes rather than causal effects of LoC per se. Another limitation is the potential for selection bias in treatment allocation. The decision for or against surgery is influenced by factors such as frailty, functional demands, or patient preferences, which cannot be fully captured in non-randomized data. Thus, residual confounding cannot be excluded despite adjustment for comorbidities and frailty measures. Finally, the presented data are explorative, and causal conclusions regarding mortality and complications cannot be drawn.

## Conclusions

Although the literature does not show a functional superiority of any treatment method for PHF in older patients, identification of treatment risks can possibly improve the outcome and quality of life. From this study, we conclude that the prior LoC has a significant impact on the course of PHF in terms of mortality, major adverse events, and thromboembolic events, as well as the choice of treatment method in older individuals. The data, once again, highlight the need for well-informed, individualized, and risk-adapted treatment recommendations to support the most vulnerable patients adequately.

## Electronic supplementary material

Below is the link to the electronic supplementary material.

Supplementary Material 1

## Data Availability

The authors state that the data utilized in this study cannot be made available in the manuscript, the additional files, or in a public repository due to German data protection laws (‘Bundesdatenschutzgesetz’, BDSG). They are stored on a server of the BARMER Institute for Health System Research, to facilitate replication of the results. In general, access to data from statutory health insurance funds for research purposes is possible only under the conditions defined in German Social Law (SGB V §287).

## Abbreviations

95% CI: 95% confidence interval
ATC: Anatomical Therapeutic Chemical Classification
HR: Hazard ratio
ICD-10 GM: International Statistical Classification of Diseases, German Modification
LoC: Level of care
LPF: Locking plate fixation after a complex fracture
LTCI: German statutory long-term care insurance
MAEs: Major adverse events
OPS: German operation and procedure classification; *Operationen- und Prozedurenschlüssel*,
OS: Overall survival
PHFs: Proximal humeral fractures
RTSA: Reverse total shoulder arthroplasty
sLPF: Locking plate fixation after a simple fracture
TEs: Thromboembolic events

## References

[1] Hasty EK, Jernigan EW, Soo A, Varkey DT, Kamath G V. Trends in surgical management and costs for operative treatment of proximal humerus fractures in the elderly. Orthopedics. 2017;40:e641–7. 28418573 10.3928/01477447-20170411-03

[2] Stolberg-Stolberg J, Köppe J, Rischen R, Freistühler M, Faldum A, Katthagen JC, et al. The surgical treatment of proximal humeral fractures in elderly patients. Dtsch Arztebl Int. 2021;118:817–23. 34730082 10.3238/arztebl.m2021.0326 PMC8888864

[3] Iglesias-Rodríguez S, Domínguez-Prado DM, García-Reza A, Fernández-Fernández D, Pérez-Alfonso E, García-Piñeiro J, et al. Epidemiology of proximal humerus fractures. J Orthop Surg Res. 2021;16:1–11. 34158100 10.1186/s13018-021-02551-x PMC8220679

[4] Launonen AP, Lepola V, Saranko A, Flinkkilä T, Laitinen M, Mattila VM. Epidemiology of proximal humerus fractures. Arch Osteoporos. 2015;10:1–5. 10.1007/s11657-015-0209-4 25675881

[5] Rupp M, Walter N, Pfeifer C, Lang S, Kerschbaum M, Krutsch W, et al. The incidence of fractures among the adult population of Germany. Dtsch Arztebl Int. 2021;118:665–69. 34140088 10.3238/arztebl.m2021.0238 PMC8727861

[6] Klug A, Gramlich Y, Wincheringer D, Schmidt-Horlohé K, Hoffmann R. Trends in surgical management of proximal humeral fractures in adults: a nationwide study of records in Germany from 2007 to 2016. Arch Orthop Trauma Surg. 2019;139:1713–21. 31375915 10.1007/s00402-019-03252-1

[7] Koeppe J, Stolberg-Stolberg J, Fischhuber K, Iking J, Marschall U, Raschke MJ. The incidence of proximal humerus fracture - an analysis of insurance data. Dtsch Arztebl Int. 2023;120:555–56. 37732593 10.3238/arztebl.m2023.0132 PMC10546881

[8] Handoll HHG, Elliott J, Thillemann TM, Aluko P, Brorson S. Interventions for treating proximal humeral fractures in adults. Cochrane Database Syst Rev. 2022, 2022. 10.1002/14651858.CD000434.pub5 PMC9211385 35727196

[9] Stolberg-Stolberg J, Köppe J, Rischen R, Freistühler M, Faldum A, Katthagen JC, et al. Influence of complications and comorbidities on length of hospital stay and costs for surgical treatment of proximal humeral fractures. Chirurg. 2021;92:907–15. 10.1007/s00104-021-01491-w PMC8463392 34533598

[10] Adam J, Basil Ammori M, Isah I, Jeyam M, Butt U. Mortality after inpatient stay for proximal humeral fractures. J Shoulder Elb Surg. 2020;29:e22–8. 10.1016/j.jse.2019.05.030 31466891

[11] Myeroff CM, Anderson JP, Sveom DS, Switzer JA. Predictors of mortality in elder patients with proximal humeral fracture. Geriatr Orthop Surg Rehabil. 2018;9. 10.1177/2151458517728155 PMC5851103 29560284

[12] Mazzucchelli RA, Jenny K, Zdravkovic V, Erhardt JB, Jost B, Spross C. The influence of local bone quality on fracture pattern in proximal humerus fractures. Injury. 2018;49:359–63. 29287662 10.1016/j.injury.2017.12.020

[13] Koeppe J, Katthagen JC, Rischen R, Freistuehler M, Faldum A, Raschke MJ, et al. Male sex is associated with higher mortality and increased risk for complications after surgical treatment of proximal humeral fractures. J Clin Med. 2021;10:2500. 34198778 10.3390/jcm10112500 PMC8201359

[14] Koeppe J, Stolberg-Stolberg J, Rischen R, Freistuehler M, Faldum A, Raschke MJ, et al. Increased complication rates of salvage reverse total shoulder arthroplasty (RTSA) after failed locked plate fixation compared with primary RTSA in the treatment of proximal humeral fractures in elderly patients. J Shoulder Elb Surg. 2023;32:1574–83. 10.1016/j.jse.2022.12.020 36682708

[15] Katthagen JC, Raschke MJ, Fischhuber K, Iking J, Marschall U, Sussiek J, et al. Conservative versus operative treatment of proximal humerus fractures in older individuals-an analysis of insurance data. Dtsch Arztebl Int. 2024;121:454–60. 38652842 10.3238/arztebl.m2024.0059 PMC11635815

[16] Hammes A, Smektala R, Halbach D, Müller-Mai C. One-year outcomes after proximal humeral fractures: a risk-adjusted regression analysis of routine data based on 17, 322 cases. Chir. 2023;94:870–76. 10.1007/s00104-023-01942-6 37608117

[17] Bundesministerium für Gesundheit. Peer review on “Germany’s latest reforms of the long-term care system” - long-term care in Germany. 2018. https://ec.europa.eu/social/BlobServlet?docId=18962%26langId=en. Accessed 5 Jun 2024.

[18] Bundesministerium für Gesundheit. Zahlen und Fakten zur Pflegeversicherung. 2023;1–20. https://www.bundesgesundheitsministerium.de/fileadmin/Dateien/3_Downloads/Statistiken/Pflegeversicherung/Zahlen_und_Fakten/Zahlen_und_Fakten_Dezember_2023.pdf. Accessed 13 Dec 2024.

[19] Köppe J, Stolberg-Stolberg J, Rischen R, Faldum A, Raschke MJ, Katthagen JC. In-hospital complications are more likely to occur after reverse Shoulder arthroplasty than after locked plating for proximal humeral fractures. Clin Orthop Relat Res. 2021;479:2284–92. 33938479 10.1097/CORR.0000000000001776 PMC8445567

[20] Gilbert T, Neuburger J, Kraindler J, Keeble E, Smith P, Ariti C, et al. Development and validation of a hospital frailty risk score focusing on older people in acute care settings using electronic hospital records: an observational study. Lancet. 2018;391:1775–82. 29706364 10.1016/S0140-6736(18)30668-8 PMC5946808

[21] Hemmann P, Ziegler P, Konrads C, Ellmerer A, Klopfer T, Schreiner AJ, et al. Trends in fracture development of the upper extremity in Germany - a population-based description of the past 15 years. J Orthop Surg Res. 2020;15:1–9. 32085794 10.1186/s13018-020-1580-4 PMC7035769

[22] Somersalo A, Paloneva J, Kautiainen H, Lönnroos E, Heinänen M, Kiviranta I. Incidence of fractures requiring inpatient care. Acta Orthop. 2014;85:525–30. 24694275 10.3109/17453674.2014.908340 PMC4164872

[23] Kandemir U, Putzeys G, McKee M. Proximal humerus fractures: treatment controversies. OTA Int. 2025;8:0–3. 10.1097/OI9.0000000000000382 PMC12045299 40321461

[24] Patel AH, Wilder JH, Ofa SA, Lee OC, Savoie FH, O’Brien MJ, et al. Trending a decade of proximal humerus fracture management in older adults. JSES Int. 2022;6:137–43. 35141688 10.1016/j.jseint.2021.08.006 PMC8811391

[25] Christensen G V, O’Reilly, Bozoghlian MF, An Q, Nepola J V, Patterson BM. Trends in the treatment of proximal humerus fractures in the United States Medicare population. Semin Arthroplast JSES. 2023;33:331–36.

[26] Cheng T, Galicia K, Patel PP, Anstadt MJ, Gonzalez RP, Kubasiak J. A nationwide analysis of geriatric proximal humerus fractures: trends, outcomes, and cost. Trauma Surg Acute Care Open. 2023;8:1–7. 10.1136/tsaco-2022-001055 PMC10391795 37533777

[27] Xu J, Sivakumar BS, Nandapalan H, Moopanar T, Harries D, Page R, et al. Trends in the surgical management of proximal humerus fractures over the last 20 years from Australian registry databases. Eur J Orthop Surg Traumatol. 2025;35. 10.1007/s00590-024-04165-5 39731653

[28] Sumrein BO, Berg HE, Launonen AP, Landell P, Laitinen MK, Felländer-Tsai L, et al. Mortality following proximal humerus fracture-a nationwide register study of 147, 692 fracture patients in Sweden. Osteoporos Int. 2023;34:349–56. 36435907 10.1007/s00198-022-06612-7 PMC9852167

[29] Brorson S, Viberg B, Gundtoft P, Jalal B, Ohrt-Nissen S. Epidemiology and trends in management of acute proximal humeral fractures in adults: an observational study of 137, 436 cases from the Danish National patient register, 1996-2018. Acta Orthop. 2022;93:750–55. 36148615 10.2340/17453674.2022.4578 PMC9500535

[30] Burkhart KJ, Dietz SO, Bastian L, Thelen U, Hoffmann R, Müller LP. Behandlung der proximalen Humerusfraktur des Erwachsenen. Dtsch Arztebl Int. 2013;110:591–97. 24078839 10.3238/arztebl.2013.0591 PMC3785018

[31] Bahrs C, Bauer M, Blumenstock G, Eingartner C, Bahrs SD, Tepass A, et al. The complexity of proximal humeral fractures is age and gender specific. J Orthop Sci. 2013;18:465–70. 23420342 10.1007/s00776-013-0361-x

[32] Shi BY, Upfill-Brown A, Kelley B V, Brodke DJ, Mayer EN, Devana SK, et al. Increasing rate of Shoulder arthroplasty for geriatric proximal humerus fractures in the United States, 2010-2019. J Shoulder Elb Arthroplast. 2022;6:247154922211371. 10.1177/24715492221137186 PMC9677166 36419867

[33] Alrabaa RG, Ma G, Truong NM, Lansdown DA, Feeley BT, Zhang AL, et al. Trends in surgical treatment of proximal humeral fractures and analysis of postoperative complications over a decade in 384, 158 patients. JBJS Open Access. 2022;7. 10.2106/JBJS.OA.22.00008 PMC9624444 36338798

[34] Shi BY, Upfill-Brown A, Wu SY, Trikha R, Ahlquist S, Kremen TJ, et al. Short-term outcomes and long-term implant survival after inpatient surgical management of geriatric proximal humerus fractures. J Shoulder Elb Arthroplast. 2023;7. 10.1177/24715492231192068 PMC10408354 37559885

[35] Neumann M V, Jaeger M, Südkamp. Komplexe proximale Humerusfrakturen: Wann ist die endoprothetische Frakturversorgung indiziert, und wenn ja, welche? Trauma und Berufskrankheit. 2017 Aug, 19;170–76.

[36] Handoll H, Brealey S, Rangan A, Keding A, Corbacho B, Jefferson L, et al. The ProFHER trial - a pragmatic multicentre randomised controlled trial evaluating the clinical effectiveness and cost-effectiveness of surgical compared with non-surgical treatment for proximal fracture of the humerus in adults. Health Technol Assess (Rockv). 2015;19:1–279. 10.3310/hta19240 PMC4781052 25822598

[37] Beks RB, Ochen Y, Frima H, Smeeing DPJ, van der Meijden O, Timmers TK, et al. Operative versus nonoperative treatment of proximal humeral fractures: a systematic review, meta-analysis, and comparison of observational studies and randomized controlled trials. J Shoulder Elb Surg. 2018;27:1526–34. 10.1016/j.jse.2018.03.009 29735376

[38] Launonen AP, Sumrein BO, Reito A, Lepola V, Paloneva J, Jonsson KB, et al. Operative versus non-operative treatment for 2-part proximal humerus fracture: a multicenter randomized controlled trial. PLoS Med. 2019;16:1–14. 10.1371/journal.pmed.1002855 PMC6638737 31318863

[39] Clement ND, Duckworth AD, McQueen MM, Court-Brown CM. The outcome of proximal humeral fractures in the elderly: predictors of mortality and function. Bone Jt J. 2014;96B: 970–77. 10.1302/0301-620X.96B7.32894 24986953

[40] Fernández-Cortiñas AB, Vidal Campos J, Marco Martínez F. Proximal humeral fracture in patients with high Charlson comorbidity index: mortality rate according to treatment choice. Musculoskelet Surg. 2021;105:167–72. 32008184 10.1007/s12306-020-00642-2

[41] Hoogendijk EO, Afilalo J, Ensrud KE, Kowal P, Onder G, Fried LP. Frailty: implications for clinical practice and public health. Lancet. 2019;394:1365–75. 31609228 10.1016/S0140-6736(19)31786-6

[42] Khoriati A, Antonios T, Bakti N, Mohanlal P, Singh B. Outcomes following non operative management for proximal humerus fractures. J Clin Orthop Trauma. 2019;10:462–67. 31061570 10.1016/j.jcot.2019.02.017 PMC6491913

