# Supplementary material for: The impact of prior level of care on the course of proximal humeral fractures in older patients: an analysis based on health insurance claims data
Source: BMC Health Serv Res. 2026 Feb 11;26:314. doi: 10.1186/s12913-026-14024-0 (PMC12937572; doi:10.1186/s12913-026-14024-0)
Supplement: Supplementary file 1 — Supplementary Material 1 [file 12913_2026_14024_MOESM1_ESM.docx]

**Additional files**

**Data availability:**

The authors confirm that the data utilized in this study cannot be made available in the manuscript, the supplemental files, or in a public repository due to German data protection laws (‘Bundesdatenschutzgesetz’, BDSG). They are stored on a server of the BARMER Institute for Health System Research, to facilitate replication of the results. In general, access to data of statutory health insurance funds for research purposes is possible only under the conditions defined in German Social Law (SGB V § 287).

**Additional file 1:** Consort flow chart. PHF – proximal humeral fracture.


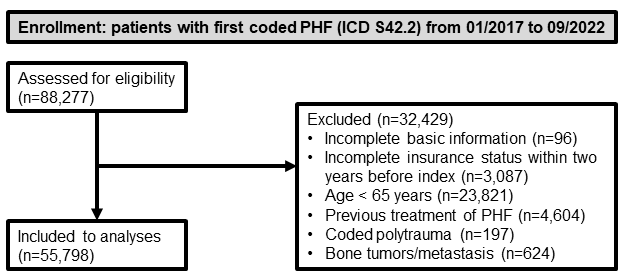


**Additional file 2:** Definition of all variables including diagnosis, procedure and pharmaceutical codes and endpoints. LPF – locked pate fixation, PHF – proximal humeral fracture, RTSA – reverse total shoulder arthroplasty.

^1^ Other forms of surgery (OPS codes): The category “other forms of surgery” includes a range of procedures that were not classified as locking plate fixation (LPF) or reverse total shoulder arthroplasty (RTSA). These OPS codes cover:

- **Open reduction without internal fixation (OPS 5-790.*)**: e.g., 5-790.01 to 5-790.91 and related suffix codes (d1, m1, n1, p1, x1). These describe open reduction of proximal humeral fractures without the use of an implant.
- **Other osteosynthesis procedures** (OPS 5-793.* and 5-794.*) including intramedullary nailing, screw fixation, cerclage wiring, and combined or less common osteosynthetic techniques (codes 5-793.11 to 5-793.x1; 5-794.01 to 5-794.x1).
- **Hemiarthroplasty and related prosthetic procedures** (OPS 5-824.*) e.g., 5-824.00, 5-824.01, 5-824.0x, 5-824.20. These codes describe partial shoulder arthroplasty procedures used in the treatment of proximal humeral fractures.

^2^ Data collection based on outpatient and inpatient information in the period of two years before the index event (i.e. first coded diagnosis of PHF). Exception: Rotator cuff rupture – only if coded at the index event.

^3^ Only based on outpatient data after PHF.

| Variable | Code | Codes |
| --- | --- | --- |
| Proximal humeral fracture | ICD | **S42.2** |
| Simple fracture LPF | OPS | **5-793.k1, 5-793.31** |
| Multi-fragmented LPF | OPS | **5-794.21, 5-794.k1** |
| RTSA | OPS | **5-824.21** |
| Other fracture fixation^1^ | OPS | **5-790.01**, 5-790.11, 5-790.21, 5-790.31, 5-790.41,  5-790.51, 5-790.61, 5-790.71, 5-790.81, 5-790.91,  5-790.d1, 5-790.m1, 5-790.n1, 5-790.p1, 5-790.x1,  **5-793.11**, 5-793.21, 5-793.41, 5-793.51, 5-793.61,  5-793.71, 5-793.81, 5-793.91, 5-793.a1, 5-793.b1,  5-793.c1, 5-793.g1, 5-793.m1, 5-793.n1, 5-793.x1,  **5-794.01**, 5-794.11, 5-794.31, 5-794.41, 5-794.51,  5-794.61, 5-794.71, 5-794.81, 5-794.a1, 5-794.b1,  5-794.c1, 5-794.g1, 5-794.m1, 5-794.n1, 5-794.x1,  **5-824.00**, 5-824.01, 5-824.0x, 5-824.20 |
| Comorbidities at index^2^ | | |
| Alcohol abuse | ICD | **E**24.4, **F**10, **G**31.2, G62.1, G72.1, **I**42.6, **K**29.2, K70, K85.2, K86.0, **T**51.0, T51.9 |
| Atherosclerosis | ICD | I70 |
| Atrial fibrillation and flutter | ICD | I48 |
| Bone tumour/metastasis | ICD | C40.0, C79.5, C79.86, C79.9 |
| Cancer | ICD | C |
| Chronic kidney disease | ICD | N18, N19 |
| Chronic polyarthritis | ICD | M05, M06 |
| Congestive heart failure | ICD | I50 |
| Coronary heart disease | ICD | I25 |
| Dementia | ICD | **F**00, F01, F02, F051, **G**30, G31.1 |
| Diabetes mellitus | ICD | E10 – E14 |
| Hypertension | ICD | I10 – I15 |
| Infection | ICD | **M**86.01, M86.11, M86.21, M86.31, M86.41, M86.51, M86.61, M86.81, M86.91, **T**84.5, T84.6, T84.7 |
| Nicotine abuses | ICD | F17 |
| Obesity | ICD | E66 |
| Osteoporosis | ICD | M80 – M85 |
| Parkinson | ICD | G20 |
| Polytrauma | ICD, OPS, DRG | ICD: T07; OPS: 5-982, or coded DRG started with “W” |
| Rotator cuff rupture | ICD | M75.1, S46.0 |
| Omarthrosis | ICD | M25.51 |
| Previous stroke and other cerebrovascular disease | ICD | I60 – I69 |
| Pharmaceutical therapy | | |
| Any anticoagulant | ATC | B01AA, B01AB, B01AC, B01AE, B01AF, B01AX |
| Vitamin D or calcium | ATC | A11CC |
| Bisphosphonates | ATC | M05BA, M05BB |
| Any osteoporosis pharmacotherapy | ATC | Vitamin D/Calcium or Bisphosphonates |
| Osteoporosis associated fractures | | |
| Distal radius | ICD | S52.2, S52.6 |
| Proximal femur | ICD | S72.0, S72.1 |
| vertebral fracture | ICD | S12.0, S12.1, S12.2, S12.7, S12.9, S22.0, S32.0 |
| pelvic ring fractures | ICD | S32.1, S32.2 S32.3, S32.5, S32.8.1 |
| Osteoporosis with pathologic fracture | ICD | M80 |
| General complications^3^ | | |
| Acute liver failure | ICD | K72.0, K72.7, K72.9 |
| Acute myocardial infarction | ICD | I21, I22 |
| Acute renal failure | ICD | N17 |
| Acute respiratory distress syndrome | ICD | J80 |
| Cardiac arrest | ICD | I46 |
| Deep vein thrombosis | ICD | I80.1, I80.2, I82.2, I82.3 |
| Pulmonary embolism | ICD | I26 |
| Sepsis | ICD | A41, A40, B37.7, R65.0, R65.1, R65.9, R57.2 |
| Stroke | ICD | I60 – I64 |
| Injured-related or surgical complications^3^ | | |
| Failure of non-surgical treatment, any | OPS | **5-78**5.01, 5-785.11, 5-785.21, 5-785.51, 5-785.61,  5-785.71, **5-789**.31, **5-78a**.01, 5-78a.11, 5-78a.21,  5-78a.31, 5-78a.41, 5-78a.51, 5-78a.61, 5-78a.71,  5-78a.90, 5-78a.c1, 5-78a.g1, 5-78a.k1, **5-790.01,**  5-790.11, 5-790.21, 5-790.31, 5-790.41, 5-790.51,  5-790.61, 5-790.71, 5-790.81, 5-790.91, 5-790.d1,  5-790.m1, 5-790.n1, 5-790.p1, 5-790.x1, **5-793**.01,  5-793.11, 5-793.21, 5-793.31, 5-793.41, 5-793.51,  5-793.81, 5-793.91, 5-793.c1, 5-793.g1, 5-793.a1,  5-793.b1, 5-793.h1, 5-793.k1, 5-793.m1, 5-793.n1,  5-793.x1, **5-794**.01, 5-794.11, 5-794.21, 5-794.31,  5-794.41, 5-794.51, 5-794.61, 5-794.71, 5-794.81,  5-794.a1, 5-794.b1, 5-794.c1, 5-794.g1, 5-794.k1,  5-794.m1, 5-794.n1, 5-794.x1, **5-824**.00**,** 5-824.01,  5-824.21, 5-824.0x |
| Upper limb amputation, ipsilateral (shoulder or upper arm) | OPS | **5-862**.1, 5-862.2 |
| Delayed union (LPF and non-surgical treatment; if coded within six months) | OPS | **5-781**.a1, **5-782**.11, 5-782.21, 5-782.31, 5-782.41,  5-782.51, 5-782.61, 5-782.a1, 5-782.b1, **5-784**.01,  5-784.11, 5-784.21, 5-784.31, 5-784.41, 5-784.71,  5-784.81, 5-784.b1 |
| Infection | OPS | **5-780**.41, 5-780.51, 5-780.61, 5-780.71, 5-780.81,  5-780.91, **5-800**.20, 5-800.30, 5-800.a0, 5-800.b0,  **5-810**.10, 5-810.70, 5-810.80, **8-989**, 8-989.0, 8-989.1, 8-989.2, 8-989.3, 8-989.4, 8-989.5, 8-989.6 |
| Joint damage / cartilage damage (LPF and non-surgical treatment) | OPS | 5-780.31, 5-784.51, 5-784.61, 5-800.80, 5-810.40,  5-812.00, 5-812.30, 5-812.90, 5-812.a0, 5-812.e0,  5-812.f0, 5-812.g0, 5-812.h0, 5-812.k0, 5-812.m0 |
| Luxation | OPS | **8-201.0** |
| Malunion (LPF and non-surgical treatment) | OPS | **5-781**.01, 5-781.11, 5-781.21, 5-781.31, 5-781.41,  5-781.51, 5-781.61, 5-781.81, 5-781.91 |
| Nerve injury | OPS | **5-040**.1, 5-040.2, 5-040.3, **5-041**.1, 5-041.2, 5-041.3,  **5-044**.1, 5-044.2, 5-044.3, **5-045**.1, 5-045.2, 5-045.3,  **5-046**.1, 5-046.2, 5-046.3, **5-047**.1, 5-047.2, 5-047.3,  **5-048**.1, 5-048.2, 5-048.3, **5-049**.1, 5-049.2, 5-049.3,  **5-04b**.1, 5-04b.2, 5-04b.3, **5-050**.1, 5-050.2, 5-050.3,  **5-051**.1, 5-051.2, 5-051.3, **5-052**.1, 5-052.2, 5-052.3,  **5-053**.1, 5-053.2, 5-053.3, **5-054**.1, 5-054.2, 5-054.3,  **5-055**.1, 5-055.2, 5-055.3, **5-056**.1, 5-056.2, 5-056.3,  **5-057**.1, 5-057.2, 5-057.3 |
| Non-union / Pseudoarthrosis (LPF and non-surgical treatment; if coded after six months) | OPS | **5-781**.a1, **5-782**.11, 5-782.21, 5-782.31, 5-782.41,  5-782.51, 5-782.61, 5-782.a1, 5-782.b1, **5-784**.01,  5-784.11, 5-784.21, 5-784.31, 5-784.41, 5-784.71,  5-784.81, 5-784.b1 |
| Osteonecrosis (LPF and non-surgical treatment) | ICD | **M87.21, M87.22, M87.32, M87.82, M87.92** |
| Postoperative stiffness, Adhesive capsulitis, Frozen shoulder | OPS | **5-800**.60, 5-800.c0, **5-810**.20, 5-810.90 |
| Vascular injury | OPS | **5-388**.11, 5-388.12, **5-395**.11, 5-395.12, **5-397**.11,  5-397.12 |
| Secondary surgery, open (LPF, non-surgical treatment) | OPS | **5-780**.01, 5-780.11, 5-780.21, 5-780.31, 5-780.61,  5-780.x1, **5-782**.11, 5-782.21, 5-782.31, 5-782.41,  5-782.51, 5-782.62, 5-782.72, 5-782.82, 5-782.92,  5-782.a1, **5-784**.01, 5-784.11, 5-784.21, 5-784.31,  5-784.41, 5-784.51, 5-784.61, 5-784.71, 5-784.81,  5-784.a1, 5-784.b1, **5-785**.01, 5-785.11, 5-785.21,  5-785.31, 5-785.41, 5-785.51, 5-785.61, 5-785.71,  **5-789**.b1, 5-789.c1, **5-794**.01, 5-794.11, 5-794.21,  5-794.31, 5-794.41, 5-794.71, 5-794.81, 5-794.k1,  **5-800**.10, 5-800.30, 5-800.40, 5-800.50, 5-800.70,  5-800.80, 5-800.90, 5-800.x0, **5-801**.00, 5-801.30,  5-801.40, 5-801.b0, 5-801.c0, 5-801.g0, 5-801.h0,  5-801.k0, 5-801.m0, 5-801.n0, 5-801.p0, **5-805**.0,  5-805.1, 5-805.2, 5-805.3, 5-805.4, 5-805.5, 5-805.6,  5-805.7, 5-805.8, 5-805.9, 5-805.a, **5-850**.01, 5-850.11, 5-850.21, 5-850.31, 5-850.41, 5-850.51, 5-850.61,  5-850.71, 5-850.81, 5-850.91, 5-850.a1, 5-850.b1,  5-850.c1, 5-850.d1, 5-850.e1, 5-850.f1, 5-850.g1,  5-850.h1, 5-850.j1, 5-850.x1, **5-851**.11, 5-851.21,  **5-852**.01, 5-852.11, **5-853**.01, 5-853.11, **5-855**.01,  5-855.11, 5-855.21, 5-855.51, 5-855.61, 5-855.71,  5-855.81, 5-855.91, 5-855.a1, **5-859**.01, 5-859.11,  **5-862**.1, 5-862.2, **5-892**.06, 5-892.07, 5-892.16,  5-892.17, **5-896**.06, 5-896.16, 5-896.26 |
| Secondary surgery, open (RTSA) | OPS | **5-780**.01, 5-780.11, 5-780.21, 5-780.31, 5-780.61,  5-780.x1, **5-782**.11, 5-782.21, 5-782.31, 5-782.41,  5-782.51, 5-782.62, 5-782.72, 5-782.82, 5-782.92,  5-782.a1, **5-785**.01, 5-785.11, 5-785.21, 5-785.31,  5-785.41, 5-785.51, 5-785.61, 5-785.71, **5-789**.b1,  **5-791**.02, 5-791.12, 5-791.22, **5-792**.02, 5-792.12,  5-792.22, 5-792.k2, **5-800**.10, 5-800.30, 5-800.40,  5-800.50, 5-800.70, 5-800.80, 5-800.90, 5-800.x0,  **5-850**.01, 5-850.11, 5-850.21, 5-850.31, 5-850.41,  5-850.51, 5-850.61, 5-850.71, 5-850.81, 5-850.91,  5-850.a1, 5-850.b1, 5-850.c1, 5-850.d1, 5-850.e1,  5-850.f1, 5-850.g1, 5-850.h1, 5-850.j1, 5-850.x1,  **5-859**.01, 5-859.11, **5-862**.1, 5-862.2, **5-892**.06,  5-892.07, 5-892.16, 5-892.17, **5-896**.06, 5-896.16,  5-896.26 |
| Secondary arthroscopy (LPF, non-surgical treatment) | OPS | **5-782**.b1, **5-784**.c1, 5-784.d1, 5-784.e1, 5-784.f1,  **5-810**.00, 5-810.10, 5-810.20, 5-810.40, 5-810.50,  5-810.70, 5-810.80, 5-810.90, **5-811**.20, 5-811.30,  5-811.40, **5-812**.00, 5-812.30, 5-812.40, 5-812.90,  5-812.a0, 5-812.e0, 5-812.f0, 5-812.g0, 5-812.h0,  5-812.k0, 5-812.m0, **5-814**.0, 5-814.1, 5-814.2, 5-814.3, 5-814.4, 5-814.5, 5-814.6, 5-814.7, 5-814.8, 5-814.9,  5-814.b, 5-814.c, 5-814.d, 5-814.e, **5-819**.00, 5-819.10, 5-819.20 |
| Secondary arthroscopy (RTSA) | OPS | **5-782**.b1, **5-810**.00, 5-810.10, 5-810.20, 5-810.40,  5-810.50, 5-810.70, 5-810.80, 5-810.90, **5-811**.20,  5-811.30, 5-811.40, **5-814**.b, 5-814.c, 5-814.d, 5-814.e, **5-819**.00, 5-819.10, 5-819.20 |
| Revision (LPF) | OPS | **5-78**5.01, 5-785.11, 5-785.21, 5-785.51, 5-785.61,  5-785.71 **5-789**.31, 5-78a.01, 5-78a.11, 5-78a.21,  5-78a.31, 5-78a.41, 5-78a.51, 5-78a.61, 5-78a.71,  5-78a.90, 5-78a.c1, 5-78a.g1, 5-78a.k1, **5-793**.01,  5-793.11, 5-793.21, 5-793.31, 5-793.41, 5-793.51,  5-793.81, 5-793.91, 5-793.c1, 5-793.g1, 5-793.a1,  5-793.b1, 5-793.h1, 5-793.k1, 5-794.01, **5-794**.11,  5-794.21, 5-794.31, 5-794.41, 5-794.71, 5-794.81,  5-794.k1, **5-824**.01, 5-824.21 |
| Revision (RTSA) | OPS | **5-785**.01, 5-785.11, **5-787**.01, 5-787.11, 5-787.21,  5-787.31, 5-787.k1, **5-789**.31, **5-78a**.01, 5-78a.11,  5-78a.21, 5-78a.k1, **5-810**.40, **5-824**.21, **5-825**.00,  5-825.1 (only 2010-2012), 5-825.21, 5-825.8, 5-825.k, 5-825.k0, 5-825.k1, 5-825.kx |
| Resection arthroplasty (for spacer placement) | OPS | **5-829.3** |
| Secondary arthroplasty (LPF and non-surgical treatment) | OPS | **5-824**.0, 5-824.20, 5-824.21 |
| Arthrolysis (LPF and non-surgical treatment) | OPS | **5-800**.60, **5-810**.20, 5-810.90 |
| Decompression of subacromial space (LPF and non-surgical treatment) | OPS | **5-814.3** |
| Debridement (LPF and non-surgical treatment) | OPS | **5-819**.10 |
| Endpoints | | |
| Major adverse events |  | resuscitation, cardiac arrest, myocardial infarction, stroke, acute renal failure, acute liver failure, acute respiratory distress syndrome, sepsis or death from any cause |
| Thromboembolic event |  | Deep vein thrombosis, pulmonary embolism, ischemic stroke or death from any cause |
| Surgical complications |  | Adhesive capsulitis, arthrolysis, conversion debridement, decompression of subacromial space, frozen shoulder, infection, infection with antibiotic-resistant germs, joint damage/cartilage damage, luxation, delayed union, non-union/ pseudoarthrosis, malunion, nerve injury, vascular injury, osteonecrosis, postoperative stiffness, secondary arthroplasty, secondary arthroscopy, secondary surgery (open) including revision surgery, upper limb amputation |
| Minor outpatient complications  (LPF) | ICD | **G**56.1, G56.2, G56.3, **I**80.80, I80.81, **M**00.01, M00.11, M00.21, M00.81, M00.91, M13.11, M13.81, M13.91, M19.11, M24.01, M24.11, M24.21, M24.41, M24.31, M24.51, M24.61, M25.11, M25.21, M25.31, M25.41, M25.51, M25.61, M25.71, M61.01, M62.21, M62.22, M62.41, M62.51, M62.61, M65.81, M65.91, M75.0, M75.1, M75.2, M75.4, M75.5, M84.21, M84.31, M86.01, M86.11, M86.21, M86.31, M86.41, M86.51, M86.61, M86.81, M86.91, M87.21, M87.22M87.31, M87.81, M87.91, M89.51, M96.6, **T**79.60, T84.10, T84.5, T84.6, T84.7 |
| Minor outpatient complications  (RTSA) | ICD | **G**56.1, G56.2, G56.3, **I**80.80, I80.81, **M**00.01, M00.11, M00.21, M00.81, M00.91, M24.21, M24.41, M24.31, M24.51, M24.61, M25.11, M25.21, M25.31, M25.41, M25.51, M25.61, M25.71, M61.01, M62.21, M62.41, M62.51, M62.61, M65.81, M65.91, M75.0, M75.1, M75.2, M75.4, M75.5, M84.31, M86.01, M86.11, M86.21, M86.31, M86.41, M86.51, M86.61, M86.81, M86.91, M96.6, **T**79.60, T81.4, T84.5, T84.6, T84.7, T84.00 |
| Minor outpatient complications  (non-surgical treatment) | ICD | **G**56.1, G56.2, G56.3, **I**80.80, I80.81, **M**00.01, M00.11, M00.21, M00.81, M00.91, M13.11, M13.81, M13.91, M19.11, M24.01, M24.11, M24.21, M24.41, M24.31, M24.51, M24.61, M25.11, M25.21, M25.31, M25.41, M25.51, M25.61, M25.71, M61.01, M62.21, M62.22, M62.41, M62.51, M62.61, M65.81, M65.91, M75.0, M75.1, M75.2, M75.4, M75.5, M84.21, M84.31, M86.01, M86.11, M86.21, M86.31, M86.41, M86.51, M86.61, M86.81, M86.91, M87.21, M87.22, M87.32, M87.82, M87.92, M89.51, M96.6, **T**79.60 |

**Additional file 3:** Results of all regression analysis. CI – confidence interval, PHF – proximal humeral fracture.

| Variable | Hazard ratio | 95%CI | P value |
| --- | --- | --- | --- |
| Overall survival – without interaction | | | |
| Year of PHF (per year) | 1.03 | 1.02 – 1.04 | <0.001 |
| Age at PHF (per year) | 1.07 | 1.06 – 1.07 | <0.001 |
| Male sex | 1.75 | 1.68 – 1.82 | <0.001 |
| Outpatient diagnosis | 0.69 | 0.66 – 0.72 | <0.001 |
| Atrial fibrillation/ flutter | 1.33 | 1.27 – 1.38 | <0.001 |
| Alcohol abuse | 1.47 | 1.37 – 1.57 | <0.001 |
| Atherosclerosis | 1.08 | 1.04 – 1.13 | <0.001 |
| Cancer | 1.14 | 1.10 – 1.18 | <0.001 |
| Congestive heart failure | 1.32 | 1.27 – 1.38 | <0.001 |
| Chronic polyarthritis | 1.07 | 1.00 – 1.14 | 0.063 |
| Chronic kidney disease | 1.23 | 1.18 – 1.28 | <0.001 |
| Dementia | 1.19 | 1.14 – 1.24 | <0.001 |
| Diabetes mellitus | 1.16 | 1.12 – 1.20 | <0.001 |
| Frozen shoulder | 0.79 | 0.71 – 0.87 | <0.001 |
| Any anticoagulant | 0.99 | 0.95 – 1.03 | 0.632 |
| Hypertension | 0.97 | 0.92 – 1.03 | 0.348 |
| Coronary heart disease | 1.03 | 0.99 – 1.07 | 0.186 |
| Nicotine abuse | 1.44 | 1.35 – 1.54 | <0.001 |
| Obesity | 0.86 | 0.83 – 0.90 | <0.001 |
| Omarthrosis | 0.86 | 0.78 – 0.95 | 0.003 |
| Any anti-osteoporotic drugs | 1.09 | 1.04 – 1.15 | <0.001 |
| Osteoporosis | 0.95 | 0.92 – 0.99 | 0.013 |
| Parkinson disease | 1.06 | 1.00 – 1.14 | 0.068 |
| Prev. stroke/other cerebrovascular disease | 0.95 | 0.91 – 0.98 | 0.008 |
| Rotator cuff rupture | 0.79 | 0.69 – 0.90 | <0.001 |
| Pre-existing nursing home care | 1.31 | 1.25 – 1.38 | <0.001 |
| Surgical treatment within 21 days (time-dependent) | 0.88 | 0.85 – 0.92 | <0.001 |
| Pre-existing Level of care V | 5.16 | 4.69 – 5.68 | <0.001 |
| Pre-existing Level of care IV | 3.89 | 3.65 – 4.15 | <0.001 |
| Pre-existing Level of care III | 2.82 | 2.67 – 2.97 | <0.001 |
| Pre-existing Level of care II | 2.23 | 2.12 – 2.35 | <0.001 |
| Pre-existing Level of care I | 1.73 | 1.55 – 1.92 | <0.001 |
| Overall survival – with interaction | | | |
| Year of PHF (per year) | 1.03 | 1.02 – 1.04 | <0.001 |
| Age at PHF (per year) | 1.07 | 1.07 – 1.07 | <0.001 |
| Male sex | 1.75 | 1.68 – 1.82 | <0.001 |
| Outpatient diagnosis | 0.69 | 0.66 – 0.72 | <0.001 |
| Atrial fibrillation/ flutter | 1.33 | 1.27 – 1.38 | <0.001 |
| Alcohol abuse | 1.47 | 1.37 – 1.57 | <0.001 |
| Atherosclerosis | 1.08 | 1.04 – 1.13 | <0.001 |
| Cancer | 1.14 | 1.10 – 1.18 | <0.001 |
| Congestive heart failure | 1.32 | 1.27 – 1.38 | <0.001 |
| Chronic polyarthritis | 1.07 | 1.00 – 1.14 | 0.063 |
| Chronic kidney disease | 1.23 | 1.19 – 1.28 | <0.001 |
| Dementia | 1.19 | 1.17 – 1.24 | <0.001 |
| Diabetes mellitus | 1.16 | 1.12 – 1.20 | <0.001 |
| Frozen shoulder | 0.77 | 0.71 – 0.87 | <0.001 |
| Any anticoagulant | 0.99 | 0.95 – 1.03 | 0.655 |
| Hypertension | 0.97 | 0.92 – 1.03 | 0.352 |
| Coronary heart disease | 1.03 | 0.99 – 1.07 | 0.188 |
| Nicotine abuse | 1.44 | 1.35 – 1.54 | <0.001 |
| Obesity | 0.86 | 0.82 – 0.90 | <0.001 |
| Omarthrosis | 0.86 | 0.78 – 0.95 | 0.004 |
| Any anti-osteoporotic drugs | 1.09 | 1.04 – 1.15 | <0.001 |
| Osteoporosis | 0.95 | 0.92 – 0.99 | 0.013 |
| Parkinson disease | 1.07 | 1.00 – 1.14 | 0.068 |
| Prev. stroke/other cerebrovascular disease | 0.95 | 0.92 – 0.99 | 0.008 |
| Rotator cuff rupture | 0.78 | 0.69 – 0.90 | <0.001 |
| Pre-existing nursing home care | 1.32 | 1.25 – 1.38 | <0.001 |
| Surgical treatment within 21 days (time-dependent) | n.a. | n.a. | <0.001 |
| Pre-existing level of care | n.a. | n.a. | <0.001 |
| Surgical treatment * level of care | n.a. | n.a. | 0.241 |
| Major adverse events – without interaction | | | |
| Year of PHF (per year) | 0.90 | 0.89 – 0.90 | <0.001 |
| Age at PHF (per year) | 1.05 | 1.05 – 1.05 | <0.001 |
| Male sex | 1.61 | 1.55 – 1.67 | <0.001 |
| Outpatient diagnosis | 0.62 | 0.60 – 0.64 | <0.001 |
| Atrial fibrillation/ flutter | 1.27 | 1.23 – 1.32 | <0.001 |
| Alcohol abuse | 1.55 | 1.46 – 1.64 | <0.001 |
| Atherosclerosis | 1.06 | 1.02 – 1.10 | 0.002 |
| Cancer | 1.08 | 1.05 – 1.11 | <0.001 |
| Congestive heart failure | 1.29 | 1.25 – 1.33 | <0.001 |
| Chronic polyarthritis | 1.09 | 1.03 – 1.15 | 0.004 |
| Chronic kidney disease | 1.33 | 1.29 – 1.38 | <0.001 |
| Dementia | 1.12 | 1.07 – 1.16 | <0.001 |
| Diabetes mellitus | 1.19 | 1.16 – 1.23 | <0.001 |
| Frozen shoulder | 0.84 | 0.78 – 0.92 | <0.001 |
| Any anticoagulant | 1.01 | 0.98 – 1.05 | 0.471 |
| Hypertension | 1.12 | 1.07 – 1.18 | <0.001 |
| Coronary heart disease | 1.04 | 1.01 – 1.08 | 0.022 |
| Nicotine abuse | 1.39 | 1.31 – 1.47 | <0.001 |
| Obesity | 0.94 | 0.90 – 0.97 | <0.001 |
| Omarthrosis | 0.87 | 0.80 – 0.94 | <0.001 |
| Any anti–osteoporotic drugs | 1.07 | 1.02 – 1.12 | 0.002 |
| Osteoporosis | 0.96 | 0.93 – 1.00 | 0.028 |
| Parkinson disease | 1.09 | 1.02 – 1.15 | 0.008 |
| Prev. stroke/other cerebrovascular disease | 1.03 | 0.99 – 1.06 | 0.130 |
| Rotator cuff rupture | 0.87 | 0.79 – 0.97 | 0.012 |
| Pre-existing nursing home care | 1.21 | 1.15 – 1.26 | <0.001 |
| Surgical treatment within 21 days (time–dependent) | 0.72 | 0.70 – 0.74 | <0.001 |
| Pre-existing Level of care V | 3.52 | 3.21 – 3.85 | <0.001 |
| Pre-existing Level of care IV | 2.83 | 2.66 – 3.00 | <0.001 |
| Pre-existing Level of care III | 2.13 | 2.03 – 2.24 | <0.001 |
| Pre-existing Level of care II | 1.84 | 1.76 – 1.92 | <0.001 |
| Pre-existing Level of care I | 1.64 | 1.50 – 1.79 | <0.001 |
| Major adverse events – with interaction | | | |
| Year of PHF (per year) | 0.89 | 0.89 – 0.90 | <0.001 |
| Age at PHF (per year) | 1.05 | 1.05 – 1.05 | <0.001 |
| Male sex | 1.61 | 1.55 – 1.67 | <0.001 |
| Outpatient diagnosis | 0.62 | 0.60 – 0.64 | <0.001 |
| Atrial fibrillation/ flutter | 1.27 | 1.23 – 1.32 | <0.001 |
| Alcohol abuse | 1.55 | 1.46 – 1.64 | <0.001 |
| Atherosclerosis | 1.06 | 1.02 – 1.10 | 0.002 |
| Cancer | 1.08 | 1.05 – 1.12 | <0.001 |
| Congestive heart failure | 1.29 | 1.25 – 1.34 | <0.001 |
| Chronic polyarthritis | 1.09 | 1.03 – 1.15 | 0.005 |
| Chronic kidney disease | 1.33 | 1.29 – 1.38 | <0.001 |
| Dementia | 1.12 | 1.07 – 1.16 | <0.001 |
| Diabetes mellitus | 1.19 | 1.16 – 1.23 | <0.001 |
| Frozen shoulder | 0.84 | 0.78 – 0.92 | <0.001 |
| Any anticoagulant | 1.01 | 0.98 – 1.05 | 0.458 |
| Hypertension | 1.12 | 1.07 – 1.18 | <0.001 |
| Coronary heart disease | 1.04 | 1.01 – 1.08 | 0.023 |
| Nicotine abuse | 1.39 | 1.31 – 1.47 | <0.001 |
| Obesity | 0.94 | 0.90 – 0.97 | <0.001 |
| Omarthrosis | 0.87 | 0.80 – 0.94 | <0.001 |
| Any anti-osteoporotic drugs | 1.07 | 1.02 – 1.12 | 0.002 |
| Osteoporosis | 0.96 | 0.93 – 1.00 | 0.028 |
| Parkinson disease | 1.09 | 1.02 – 1.15 | 0.007 |
| Prev. stroke/other cerebrovascular disease | 1.03 | 1.00 – 1.06 | 0.063 |
| Rotator cuff rupture | 0.87 | 0.79 – 0.97 | 0.011 |
| Pre-existing nursing home care | 1.20 | 1.15 – 1.26 | <0.001 |
| Surgical treatment within 21 days (time-dependent) | n.a. | n.a. | <0.001 |
| Pre-existing Level of care | n.a. | n.a. | <0.001 |
| Surgical treatment * Level of care | n.a. | n.a. | 0.139 |
| Thromboembolic events (or death) – without interaction | | | |
| Year of PHF (per year) | 0.89 | 0.88 – 0.90 | <0.001 |
| Age at PHF (per year) | 1.06 | 1.05 – 1.06 | <0.001 |
| Male sex | 1.64 | 1.57 – 1.70 | <0.001 |
| Outpatient diagnosis | 0.64 | 0.62 – 0.66 | <0.001 |
| Atrial fibrillation/ flutter | 1.25 | 1.20 – 1.30 | <0.001 |
| Alcohol abuse | 1.39 | 1.30 – 1.48 | <0.001 |
| Atherosclerosis | 1.07 | 1.03 – 1.11 | <0.001 |
| Cancer | 1.11 | 1.07 – 1.14 | <0.001 |
| Congestive heart failure | 1.26 | 1.21 – 1.31 | <0.001 |
| Chronic polyarthritis | 1.08 | 1.02 – 1.15 | 0.015 |
| Chronic kidney disease | 1.21 | 1.17 – 1.25 | <0.001 |
| Dementia | 1.17 | 1.12 – 1.22 | <0.001 |
| Diabetes mellitus | 1.16 | 1.12 – 1.20 | <0.001 |
| Frozen shoulder | 0.82 | 0.75 – 0.90 | <0.001 |
| Any anticoagulant | 1.00 | 0.96 – 1.04 | 0.986 |
| Hypertension | 1.03 | 0.98 – 1.09 | 0.249 |
| Coronary heart disease | 1.01 | 0.98 – 1.05 | 0.438 |
| Nicotine abuse | 1.37 | 1.29 – 1.46 | <0.001 |
| Obesity | 0.90 | 0.86 – 0.93 | <0.001 |
| Omarthrosis | 0.91 | 0.83 – 0.99 | 0.029 |
| Any anti–osteoporotic drugs | 1.07 | 1.02 – 1.12 | 0.004 |
| Osteoporosis | 0.98 | 0.95 – 1.01 | 0.236 |
| Parkinson disease | 1.07 | 1.00 – 1.14 | 0.053 |
| Prev. stroke/other cerebrovascular disease | 1.01 | 0.98 – 1.04 | 0.603 |
| Rotator cuff rupture | 0.83 | 0.74 – 0.93 | 0.002 |
| Pre-existing nursing home care | 1.26 | 1.20 – 1.32 | <0.001 |
| Surgical treatment within 21 days (time–dependent) | 0.69 | 0.67 – 0.71 | <0.001 |
| Pre-existing Level of care V | 4.02 | 3.66 – 4.41 | <0.001 |
| Pre-existing Level of care IV | 3.09 | 2.90 – 3.28 | <0.001 |
| Pre-existing Level of care III | 2.33 | 2.21 – 2.45 | <0.001 |
| Pre-existing Level of care II | 1.90 | 1.81 – 2.00 | <0.001 |
| Pre-existing Level of care I | 1.60 | 1.45 – 1.76 | <0.001 |
| Thromboembolic events (or death) – with interaction | | | |
| Year of PHF (per year) | 0.89 | 0.88 – 0.90 | <0.001 |
| Age at PHF (per year) | 1.06 | 1.05 – 1.06 | <0.001 |
| Male sex | 1.64 | 1.57 – 1.70 | <0.001 |
| Outpatient diagnosis | 0.64 | 0.62 – 0.67 | <0.001 |
| Atrial fibrillation/ flutter | 1.25 | 1.20 – 1.29 | <0.001 |
| Alcohol abuse | 1.39 | 1.30 – 1.48 | <0.001 |
| Atherosclerosis | 1.07 | 1.03 – 1.11 | <0.001 |
| Cancer | 1.11 | 1.07 – 1.14 | <0.001 |
| Congestive heart failure | 1.26 | 1.22 – 1.31 | <0.001 |
| Chronic polyarthritis | 1.08 | 1.02 – 1.15 | 0.015 |
| Chronic kidney disease | 1.21 | 1.17 – 1.25 | <0.001 |
| Dementia | 1.17 | 1.12 – 1.22 | <0.001 |
| Diabetes mellitus | 1.16 | 1.12 – 1.20 | <0.001 |
| Frozen shoulder | 0.82 | 0.75 – 0.90 | <0.001 |
| Any anticoagulant | 1.00 | 0.96 – 1.04 | >0.999 |
| Hypertension | 1.03 | 0.98 – 1.09 | 0.248 |
| Coronary heart disease | 1.01 | 0.98 – 1.05 | 0.441 |
| Nicotine abuse | 1.37 | 1.29 – 1.46 | <0.001 |
| Obesity | 0.90 | 0.86 – 0.93 | <0.001 |
| Omarthrosis | 0.91 | 0.83 – 0.99 | 0.030 |
| Any anti-osteoporotic drugs | 1.07 | 1.02 – 1.12 | 0.004 |
| Osteoporosis | 0.98 | 0.95 – 1.01 | 0.241 |
| Parkinson disease | 1.07 | 1.00 – 1.14 | 0.052 |
| Prev. stroke/other cerebrovascular disease | 1.01 | 0.98 – 1.04 | 0.599 |
| Rotator cuff rupture | 0.83 | 0.74 – 0.93 | 0.001 |
| Pre-existing nursing home care | 1.26 | 1.20 – 1.32 | <0.001 |
| Surgical treatment within 21 days (time-dependent) | n.a. | n.a. | <0.001 |
| Pre-existing Level of care | n.a. | n.a. | <0.001 |
| Surgical treatment * Level of care | n.a. | n.a. | 0.556 |
| Secondary osteoporosis-associated fractures | | | |
| Year of PHF (per year) | 0.94 | 0.93 – 0.96 | <0.001 |
| Age at PHF (per year) | 1.03 | 1.03 – 1.04 | <0.001 |
| Male sex | 0.83 | 0.77 – 0.89 | <0.001 |
| Outpatient diagnosis | 0.82 | 0.78 – 0.87 | <0.001 |
| Atrial fibrillation/ flutter | 1.00 | 0.93 – 1.07 | 0.948 |
| Alcohol abuse | 1.34 | 1.21 – 1.49 | <0.001 |
| Atherosclerosis | 1.06 | 1.00 – 1.13 | 0.050 |
| Cancer | 1.00 | 0.95 – 1.05 | 0.895 |
| Congestive heart failure | 0.96 | 0.90 – 1.02 | 0.161 |
| Chronic polyarthritis | 1.06 | 0.97 – 1.16 | 0.238 |
| Chronic kidney disease | 0.96 | 0.90 – 1.01 | 0.121 |
| Dementia | 1.21 | 1.12 – 1.30 | <0.001 |
| Diabetes mellitus | 0.99 | 0.94 – 1.05 | 0.792 |
| Frozen shoulder | 0.93 | 0.83 – 1.05 | 0.259 |
| Any anticoagulant | 1.06 | 1.00 – 1.13 | 0.058 |
| Hypertension | 1.04 | 0.97 – 1.11 | 0.270 |
| Coronary heart disease | 0.96 | 0.91 – 1.02 | 0.212 |
| Nicotine abuse | 1.29 | 1.18 – 1.41 | <0.001 |
| Obesity | 0.84 | 0.79 – 0.90 | <0.001 |
| Omarthrosis | 0.93 | 0.81 – 1.06 | 0.271 |
| Parkinson disease | 1.52 | 1.37 – 1.69 | <0.001 |
| Prev. stroke/other cerebrovascular disease | 1.11 | 1.05 – 1.17 | <0.001 |
| Rotator cuff rupture | 0.97 | 0.84 – 1.13 | 0.726 |
| Pre-existing nursing home care | 0.75 | 0.68 – 0.83 | <0.001 |
| Surgical treatment within 21 days (time–dependent) | 0.73 | 0.70 – 0.78 | <0.001 |
| Pre-existing Level of care V | 0.40 | 0.30 – 0.53 | <0.001 |
| Pre-existing Level of care IV | 0.73 | 0.64 – 0.82 | <0.001 |
| Pre-existing Level of care III | 0.92 | 0.84 – 1.00 | 0.062 |
| Pre-existing Level of care II | 1.01 | 0.93 – 1.09 | 0.858 |
| Pre-existing Level of care I | 1.10 | 0.95 – 1.27 | 0.188 |
| Osteoporosis incl. FU (time dependent) | 1.74 | 1.64 – 1.84 | <0.001 |
| Any anti-osteoporotic drugs incl. FU (time dependent) | 0.68 | 0.64 – 0.73 | <0.001 |
| Minor outpatient complication – without interaction | | | |
| Year of PHF (per year) | 1.00 | 0.99 – 1.01 | 0.383 |
| Age at PHF (per year) | 0.98 | 0.98 – 0.98 | <0.001 |
| Male sex | 0.98 | 0.94 – 1.03 | 0.461 |
| Outpatient diagnosis | 1.14 | 1.10 – 1.18 | <0.001 |
| Atrial fibrillation/ flutter | 0.88 | 0.84 – 0.93 | <0.001 |
| Alcohol abuse | 0.97 | 0.90 – 1.04 | 0.376 |
| Atherosclerosis | 1.05 | 1.01 – 1.10 | 0.021 |
| Cancer | 1.01 | 0.98 – 1.05 | 0.436 |
| Congestive heart failure | 0.95 | 0.91 – 0.99 | 0.020 |
| Chronic polyarthritis | 1.08 | 1.02 – 1.15 | 0.010 |
| Chronic kidney disease | 1.01 | 0.97 – 1.05 | 0.692 |
| Dementia | 0.83 | 0.78 – 0.89 | <0.001 |
| Diabetes mellitus | 0.97 | 0.94 – 1.01 | 0.126 |
| Frozen shoulder | 3.08 | 2.90 – 3.28 | <0.001 |
| Any anticoagulant | 0.98 | 0.94 – 1.03 | 0.456 |
| Hypertension | 1.08 | 1.03 – 1.13 | <0.001 |
| Coronary heart disease | 1.09 | 1.04 – 1.13 | <0.001 |
| Nicotine abuse | 0.94 | 0.88 – 1.00 | 0.033 |
| Obesity | 1.15 | 1.11 – 1.20 | <0.001 |
| Omarthrosis | 2.24 | 2.08 – 2.40 | <0.001 |
| Any anti–osteoporotic drugs | 1.07 | 1.02 – 1.13 | 0.005 |
| Osteoporosis | 1.15 | 1.11 – 1.19 | <0.001 |
| Parkinson disease | 1.09 | 1.00 – 1.20 | 0.064 |
| Prev. stroke/other cerebrovascular disease | 1.11 | 1.07 – 1.16 | <0.001 |
| Rotator cuff rupture | 3.42 | 3.18 – 3.67 | <0.001 |
| Pre-existing nursing home care | 0.69 | 0.62 – 0.77 | <0.001 |
| Surgical treatment within 21 days (time–dependent) | 0.91 | 0.88 – 0.95 | <0.001 |
| Pre-existing Level of care V | 0.36 | 0.28 – 0.46 | <0.001 |
| Pre-existing Level of care IV | 0.52 | 0.46 – 0.58 | <0.001 |
| Pre-existing Level of care III | 0.61 | 0.56 – 0.66 | <0.001 |
| Pre-existing Level of care II | 0.78 | 0.73 – 0.83 | <0.001 |
| Pre-existing Level of care I | 0.87 | 0.78 – 0.97 | 0.009 |
| Minor outpatient complication – with interaction | | | |
| Year of PHF (per year) | 1.00 | 0.99 – 1.01 | 0.426 |
| Age at PHF (per year) | 0.98 | 0.98 – 0.98 | <0.001 |
| Male sex | 0.98 | 0.94 – 1.03 | 0.475 |
| Outpatient diagnosis | 1.15 | 1.11 – 1.19 | <0.001 |
| Atrial fibrillation/ flutter | 0.88 | 0.84 – 0.93 | <0.001 |
| Alcohol abuse | 0.97 | 0.90 – 1.04 | 0.382 |
| Atherosclerosis | 1.05 | 1.01 – 1.10 | 0.021 |
| Cancer | 1.02 | 0.98 – 1.05 | 0.424 |
| Congestive heart failure | 0.95 | 0.91 – 0.99 | 0.022 |
| Chronic polyarthritis | 1.08 | 1.02 – 1.15 | 0.009 |
| Chronic kidney disease | 1.01 | 0.97 – 1.05 | 0.689 |
| Dementia | 0.83 | 0.78 – 0.89 | <0.001 |
| Diabetes mellitus | 0.97 | 0.94 – 1.01 | 0.128 |
| Frozen shoulder | 3.08 | 2.90 – 3.28 | <0.001 |
| Any anticoagulant | 0.98 | 0.94 – 1.03 | 0.438 |
| Hypertension | 1.08 | 1.03 – 1.13 | <0.001 |
| Coronary heart disease | 1.09 | 1.04 – 1.13 | <0.001 |
| Nicotine abuse | 0.94 | 0.88 – 1.00 | 0.033 |
| Obesity | 1.15 | 1.11 – 1.20 | <0.001 |
| Omarthrosis | 2.24 | 2.08 – 2.40 | <0.001 |
| Any anti-osteoporotic drugs | 1.08 | 1.02 – 1.13 | 0.004 |
| Osteoporosis | 1.15 | 1.11 – 1.19 | <0.001 |
| Parkinson disease | 1.09 | 1.00 – 1.20 | 0.065 |
| Prev. stroke/other cerebrovascular disease | 1.11 | 1.07 – 1.15 | <0.001 |
| Rotator cuff rupture | 3.43 | 3.19 – 3.68 | <0.001 |
| Pre-existing nursing home care | 0.69 | 0.62 – 0.76 | <0.001 |
| Surgical treatment within 21 days (time-dependent) | n.a. | n.a. | <0.001 |
| Pre-existing Level of care | n.a. | n.a. | <0.001 |
| Surgical treatment * Level of care | n.a. | n.a. | <0.001 |
| Surgical/injured-related complications – without interaction | | | |
| Year of PHF (per year) | 0.97 | 0.95 – 0.99 | 0.002 |
| Age at PHF (per year) | 0.98 | 0.97 – 0.98 | <0.001 |
| Male sex | 1.10 | 1.02 – 1.20 | 0.020 |
| Outpatient diagnosis | 0.72 | 0.67 – 0.77 | <0.001 |
| Atrial fibrillation/ flutter | 0.99 | 0.91 – 1.09 | 0.889 |
| Alcohol abuse | 1.42 | 1.26 – 1.59 | <0.001 |
| Atherosclerosis | 0.98 | 0.90 – 1.06 | 0.614 |
| Cancer | 1.02 | 0.96 – 1.09 | 0.531 |
| Congestive heart failure | 1.05 | 0.97 – 1.14 | 0.231 |
| Chronic polyarthritis | 1.13 | 1.01 – 1.26 | 0.035 |
| Chronic kidney disease | 1.06 | 0.99 – 1.14 | 0.112 |
| Dementia | 0.98 | 0.87 – 1.09 | 0.688 |
| Diabetes mellitus | 1.04 | 0.97 – 1.11 | 0.280 |
| Frozen shoulder | 1.19 | 1.03 – 1.36 | 0.015 |
| Any anticoagulant | 1.07 | 0.99 – 1.16 | 0.091 |
| Hypertension | 1.17 | 1.07 – 1.28 | <0.001 |
| Coronary heart disease | 1.00 | 0.93 – 1.08 | 0.909 |
| Nicotine abuse | 1.05 | 0.94 – 1.16 | 0.403 |
| Obesity | 1.21 | 1.12 – 1.30 | <0.001 |
| Omarthrosis | 1.10 | 0.94 – 1.29 | 0.224 |
| Any anti–osteoporotic drugs | 1.09 | 1.00 – 1.20 | 0.055 |
| Osteoporosis | 1.10 | 1.03 – 1.18 | 0.008 |
| Parkinson disease | 1.34 | 1.16 – 1.56 | <0.001 |
| Prev. stroke/other cerebrovascular disease | 0.94 | 0.88 – 1.01 | 0.111 |
| Rotator cuff rupture | 1.40 | 1.21 – 1.63 | <0.001 |
| Pre-existing nursing home care | 0.73 | 0.62 – 0.86 | <0.001 |
| Surgical treatment within 21 days (time–dependent) | 1.35 | 1.26 – 1.44 | <0.001 |
| Pre-existing Level of care V | 0.37 | 0.23 – 0.59 | <0.001 |
| Pre-existing Level of care IV | 0.65 | 0.54 – 0.79 | <0.001 |
| Pre-existing Level of care III | 0.88 | 0.77 – 1.00 | 0.041 |
| Pre-existing Level of care II | 0.92 | 0.83 – 1.02 | 0.124 |
| Pre-existing Level of care I | 1.09 | 0.91 – 1.30 | 0.366 |
| Surgical/injured-related complications – with interaction | | | |
| Year of PHF (per year) | 0.97 | 0.95 – 0.99 | 0.002 |
| Age at PHF (per year) | 0.98 | 0.97 – 0.98 | <0.001 |
| Male sex | 1.10 | 1.02 – 1.20 | 0.020 |
| Outpatient diagnosis | 0.72 | 0.67 – 0.77 | <0.001 |
| Atrial fibrillation/ flutter | 0.99 | 0.91 – 1.09 | 0.877 |
| Alcohol abuse | 1.42 | 1.26 – 1.59 | <0.001 |
| Atherosclerosis | 0.98 | 0.90 – 1.06 | 0.604 |
| Cancer | 1.02 | 0.96 – 1.09 | 0.538 |
| Congestive heart failure | 1.05 | 0.97 – 1.14 | 0.237 |
| Chronic polyarthritis | 1.13 | 1.01 – 1.26 | 0.035 |
| Chronic kidney disease | 1.06 | 0.99 – 1.14 | 0.112 |
| Dementia | 0.98 | 0.87 – 1.09 | 0.675 |
| Diabetes mellitus | 1.04 | 0.97 – 1.11 | 0.283 |
| Frozen shoulder | 1.19 | 1.03 – 1.36 | 0.015 |
| Any anticoagulant | 1.07 | 0.99 – 1.16 | 0.091 |
| Hypertension | 1.17 | 1.07 – 1.28 | <0.001 |
| Coronary heart disease | 1.00 | 0.93 – 1.08 | 0.923 |
| Nicotine abuse | 1.05 | 0.94 – 1.16 | 0.400 |
| Obesity | 1.21 | 1.12 – 1.30 | <0.001 |
| Omarthrosis | 1.10 | 0.94 – 1.29 | 0.225 |
| Any anti-osteoporotic drugs | 1.09 | 1.00 – 1.20 | 0.057 |
| Osteoporosis | 1.10 | 1.03 – 1.18 | 0.008 |
| Parkinson disease | 1.34 | 1.16 – 1.56 | <0.001 |
| Prev. stroke/other cerebrovascular disease | 0.94 | 0.88 – 1.01 | 0.112 |
| Rotator cuff rupture | 1.40 | 1.21 – 1.63 | <0.001 |
| Pre-existing nursing home care | 0.73 | 0.62 – 0.87 | <0.001 |
| Surgical treatment within 21 days (time-dependent) | n.a. | n.a. | <0.001 |
| Pre-existing Level of care | n.a. | n.a. | <0.001 |
| Surgical treatment * Level of care | n.a. | n.a. | 0.552 |
| Surgical/injured-related complications – time dependent without interaction | | | |
| Year of PHF (per year) | 0.97 | 0.95 – 0.99 | 0.005 |
| Age at PHF (per year) | 0.98 | 0.97 – 0.98 | <0.001 |
| Male sex | 1.11 | 1.02 – 1.20 | 0.016 |
| Outpatient diagnosis | 0.72 | 0.67 – 0.78 | <0.001 |
| Atrial fibrillation/ flutter | 1.00 | 0.91 – 1.09 | 0.904 |
| Alcohol abuse | 1.42 | 1.27 – 1.60 | <0.001 |
| Atherosclerosis | 0.98 | 0.91 – 1.06 | 0.629 |
| Cancer | 1.02 | 0.96 – 1.09 | 0.512 |
| Congestive heart failure | 1.05 | 0.97 – 1.14 | 0.214 |
| Chronic polyarthritis | 1.13 | 1.01 – 1.26 | 0.035 |
| Chronic kidney disease | 1.06 | 0.99 – 1.15 | 0.097 |
| Dementia | 0.98 | 0.88 – 1.10 | 0.732 |
| Diabetes mellitus | 1.04 | 0.97 – 1.11 | 0.273 |
| Frozen shoulder | 1.19 | 1.03 – 1.36 | 0.015 |
| Any anticoagulant | 1.07 | 0.99 – 1.16 | 0.086 |
| Hypertension | 1.17 | 1.07 – 1.28 | <0.001 |
| Coronary heart disease | 1.00 | 0.93 – 1.08 | 0.915 |
| Nicotine abuse | 1.05 | 0.94 – 1.17 | 0.392 |
| Obesity | 1.21 | 1.12 – 1.30 | <0.001 |
| Omarthrosis | 1.10 | 0.94 – 1.29 | 0.227 |
| Any anti–osteoporotic drugs | 1.10 | 1.00 – 1.20 | 0.054 |
| Osteoporosis | 1.10 | 1.02 – 1.18 | 0.008 |
| Parkinson disease | 1.35 | 1.16 – 1.56 | <0.001 |
| Prev. stroke/other cerebrovascular disease | 0.94 | 0.88 – 1.01 | 0.112 |
| Rotator cuff rupture | 1.40 | 1.20 – 1.63 | <0.001 |
| Pre-existing nursing home care | 0.73 | 0.62 – 0.87 | <0.001 |
| Surgical treatment up to 3 months after PHF | 1.29 | 1.18 – 1.40 | <0.001 |
| Surgical treatment 3 – 6 months after PHF | 1.86 | 1.58 – 2.20 | <0.001 |
| Surgical treatment 6 – 12 months after PHF | 2.22 | 1.87 – 2.63 | <0.001 |
| Surgical treatment 1 – 2 years after PHF | 1.38 | 1.15 – 1.66 | <0.001 |
| Surgical treatment 2 – 3 years after PHF | 0.52 | 0.39 – 0.69 | <0.001 |
| Surgical treatment 3 – 4 years after PHF | 0.40 | 0.26 – 0.62 | <0.001 |
| Surgical treatment 4 –5 years after PHF | 0.25 | 0.14 – 0.48 | <0.001 |
| Surgical treatment 5 – 6 years after PHF | 0.20 | 0.05 – 0.77 | 0.019 |
| Surgical treatment >6 years after PHF | 0.38 | 0.24 – 0.60 | <0.001 |
| Pre-existing Level of care V | 0.38 | 0.24 – 0.60 | <0.001 |
| Pre-existing Level of care IV | 0.66 | 0.55 – 0.80 | <0.001 |
| Pre-existing Level of care III | 0.89 | 0.78 – 1.00 | 0.058 |
| Pre-existing Level of care II | 0.92 | 0.83 – 1.03 | 0.137 |
| Pre-existing Level of care I | 1.09 | 0.91 – 1.31 | 0.354 |
| Surgical/injured-related complications – time dependent with interaction | | | |
| Year of PHF (per year) | 0.97 | 0.96 – 0.99 | 0.008 |
| Age at PHF (per year) | 0.98 | 0.97 – 0.98 | <0.001 |
| Male sex | 1.11 | 1.02 – 1.21 | 0.012 |
| Outpatient diagnosis | 0.73 | 0.68 – 0.78 | <0.001 |
| Atrial fibrillation/ flutter | 1.00 | 0.91 – 1.09 | 0.998 |
| Alcohol abuse | 1.41 | 1.26 – 1.59 | <0.001 |
| Atherosclerosis | 0.98 | 0.91 – 1.06 | 0.635 |
| Cancer | 1.02 | 0.96 – 1.09 | 0.510 |
| Congestive heart failure | 1.05 | 0.97 – 1.14 | 0.198 |
| Chronic polyarthritis | 1.13 | 1.01 – 1.26 | 0.035 |
| Chronic kidney disease | 1.07 | 0.99 – 1.15 | 0.085 |
| Dementia | 0.98 | 0.88 – 1.10 | 0.737 |
| Diabetes mellitus | 1.04 | 0.97 – 1.11 | 0.291 |
| Frozen shoulder | 1.19 | 1.04 – 1.36 | 0.015 |
| Any anticoagulant | 1.07 | 0.99 – 1.16 | 0.095 |
| Hypertension | 1.17 | 1.07 – 1.28 | <0.001 |
| Coronary heart disease | 1.00 | 0.93 – 1.08 | 0.916 |
| Nicotine abuse | 1.05 | 0.94 – 1.17 | 0.383 |
| Obesity | 1.21 | 1.12 – 1.30 | <0.001 |
| Omarthrosis | 1.10 | 0.94 – 1.29 | 0.215 |
| Any anti–osteoporotic drugs | 1.09 | 1.00 – 1.20 | 0.058 |
| Osteoporosis | 1.10 | 1.02 – 1.18 | 0.009 |
| Parkinson disease | 1.33 | 1.15 – 1.55 | <0.001 |
| Prev. stroke/other cerebrovascular disease | 0.94 | 0.88 – 1.01 | 0.107 |
| Rotator cuff rupture | 1.39 | 1.19 – 1.62 | <0.001 |
| Pre-existing nursing home care | 0.76 | 0.64 – 0.90 | 0.002 |
| Surgical treatment up to 3 months after PHF | n.a. | n.a. | 0.095 |
| Surgical treatment 3 – 6 months after PHF | n.a. | n.a. | <0.001 |
| Surgical treatment 6 – 12 months after PHF | n.a. | n.a. | <0.001 |
| Surgical treatment 1 – 2 years after PHF | n.a. | n.a. | <0.001 |
| Surgical treatment 2 – 3 years after PHF | n.a. | n.a. | 0.002 |
| Surgical treatment 3 – 4 years after PHF | n.a. | n.a. | 0.007 |
| Surgical treatment 4 –5 years after PHF | n.a. | n.a. | 0.001 |
| Surgical treatment >5 years after PHF | n.a. | n.a. | 0.069 |
| Pre-existing Level of care (LoC) | n.a. | n.a. | <0.001 |
| Surgical treatment up to 3 months after PHF*LoC | n.a. | n.a. | 0.958 |
| Surgical treatment 3 – 6 months after PHF*LoC | n.a. | n.a. | <0.001 |
| Surgical treatment 6 – 12 months after PHF*LoC | n.a. | n.a. | <0.001 |
| Surgical treatment 1 – 2 years after PHF*LoC | n.a. | n.a. | <0.001 |
| Surgical treatment 2 – 3 years after PHF*LoC | n.a. | n.a. | <0.001 |
| Surgical treatment 3 – 4 years after PHF*LoC | n.a. | n.a. | <0.001 |
| Surgical treatment 4 –5 years after PHF*LoC | n.a. | n.a. | <0.001 |
| Surgical treatment >5 years after PHF*LoC | n.a. | n.a. | <0.001 |

**Additional file 4**: Cumulative incidence functions/event rates (95% confidence interval) for any surgical or injured related complications, major adverse events (MAEs), thromboembolic events or death and overall mortality determined by Aalen-Johansen estimates. For any surgical event, death was considered as a competing risk event. Patients were classified based on preexisting Level of Care (LoC). major adverse event – MAE, proximal humeral fracture – PHF, surgical complications – SC.

|  | Entire Cohort | No LoC | LoC I | LoC II | LoC III | LoC IV | LoC V |
| --- | --- | --- | --- | --- | --- | --- | --- |
| Overall mortality  1 year rate  2 years rate  3 years rate  4 years rate  5 years rate  total no. of events | 11.6%  (11.4 – 11.9%)  18.2%  (17.9 – 18.6%)  24.6%  (24.2 – 25.0%)  30.7%  (30.2 – 31.2%)  36.4%  (35.8 – 37.0%)  13,790 | 4.4%  (4.2 – 4.6%)  7.8%  (7.5 – 8.0%)  11.8%  (11.4 – 12.2%)  16.3%  (15.8 – 16.8%)  21.2%  (20.5 – 21.8%)  4,942 | 11.7%  (10.0% – 13.5%)  21.7%  (19.3 – 24.2%)  30.5%  (27.5 – 33.5%)  39.7%  (35.8 – 43.6%)  49.5%  (43.4 – 55.3%)  364 | 19.3%  (18.4 – 20.3%)  31.0%  (29.8 – 32.2%)  42.5%  (41.0 – 43.9%)  53.6%  (52.0 – 55.3%)  62.6%  (60.6 – 64.5%)  2,672 | 28.6%  (27.4 – 29.8%)  43.7%  (42.3 – 45.0%)  56.7%  (55.2 – 58.2%)  67.3%  (65.6 – 68.9%)  76.2%  (74.2 – 78.0%)  3,098 | 41.2%  (39.4 – 42.9%)  58.2%  (56.3 – 60.0%)  70.6%  (68.7 – 72.5%)  79.6%  (77.6 – 81.4%)  86.4%  (84.3 – 88.3%)  2,121 | 49.6%  (46.0 – 53.0%)  65.2%  (61.5 – 68.5%)  76.3%  (72.7 – 79.4%)  84.8%  (81.2 – 87.7%)  88.6%  (84.7 – 91.6%)  593 |
| MAEs  1 year rate  2 years rate  3 years rate  4 years rate  5 years rate  total no. of events | 17.3%  (16.9 – 17.6%)  24.1%  (23.8 – 24.5%)  30.0%  (29.7 – 30.5%)  35.0%  (34.5 – 35.4%)  38.7%  (38.2 – 39.2%)  18,446 | 8.9%  (8.6 – 9.2%)  13.4%  (13.1 – 13.8%)  17.9%  (17.5 – 18.3%)  22.1%  (21.7 – 22.6%)  25.7%  (25.2 – 26.2%)  8,055 | 20.6%  (18.5 – 22.8%)  31.1%  (28.6 – 33.6%)  38.6%  (35.8 – 41.3%)  44.9%  (41.7 – 48.0%)  49.3%  (45.6 – 52.9%)  544 | 28.4%  (27.3 – 29.5%)  39.4%  (38.2 – 40.6%)  48.7%  (47.4 – 50.0%)  56.2%  (54.8 – 57.6%)  61.3%  (59.8 – 62.9%)  3,362 | 36.0%  (34.8 – 37.3%)  48.7%  (47.4 – 50.0%)  59.1%  (57.8 – 60.5%)  66.4%  (64.9 – 67.8%)  71.3%  (69.7 – 72.9%)  3,548 | 47.3%  (45.5 – 49.0%)  61.0%  (59.3 – 62.7%)  71.0%  (69.3 – 72.7%)  77.8%  (75.9 – 79.5%)  82.6%  (80.6 – 84.4%)  2,313 | 54.7%  (51.2 – 58.0%)  66.9%  (63.4 – 70.0%)  76.0%  (72.6 – 79.0%)  82.6%  (79.2 – 85.4%)  85.6%  (82.1 – 88.5%)  624 |
| Thromboembolic events (or death)  1 year rate  2 years rate  3 years rate  4 years rate  5 years rate  total no. of events | 13.9%  (13.6 – 14.2%)  20.2%  (19.8 – 20.5%)  25.7%  (25.4 – 26.1%)  30.6%  (30.2 – 31.0%)  34.3%  (33.8 – 34.8%)  16,086 | 6.5%  (6.3 – 6.8%)  10.3%  (10.00 – 10.6%)  14.2%  (13.8 – 14.6%)  18.1%  (17.6 – 18.5%)  21.4%  (20.9 – 21.9%)  6,595 | 14.6%  (12.8 – 16.5%)  23.6%  (21.3 – 25.9%)  30.8%  (28.1 – 33.4%)  37.0%  (34.0 – 40.1%)  41.9%  (38.2 – 45.6%)  440 | 22.3%  (21.3 – 23.3%)  32.5%  (31.4 – 33.7%)  41.96%  (40.7 – 43.2%)  50.1%  (48.6 – 51.5%)  55.2%  (53.6 – 56.7%)  2,956 | 30.8%  (29.6 – 32.0%)  43.7%  (42.4 – 45.0%)  53.9%  (52.5 – 55.3%)  62.00%  (60.5 – 63.4%)  67.9%  (66.2 – 69.6%)  3,296 | 42.5%  (40.8 – 44.2%)  56.6%  (54.8 – 58.3%)  66.9%  (65.1 – 68.6%)  74.2%  (72.3 – 76.0%)  79.1%  (77.0 – 81.0%)  2,192 | 51.2%  (47.7 – 54.6%)  64.1%  (60.7 – 67.4%)  74.1%  (70.7 – 77.2%)  80.9%  (77.5 – 83.9%)  83.7%  (80.0 – 86.7%)  607 |
| SC starting 21 days after PHF  1 year rate  2 years rate  3 years rate  4 years rate  5 years rate  total no. of events | 6.5%  (6.3 – 6.7%)  7.7%  (7.4 – 7.9%)  8.2%  (8.0 – 8.5%)  8.6%  (8.3 – 8.8%)  8.9%  (8.7 – 9.2%)  4,399 | 6.9%  (6.7 – 7.2%)  8.3%  (8.0 – 8.6%)  9.0%  (8.7 – 9.3%)  9.4%  (9.0 – 9.7%)  9.8%  (9.5 – 10.1%)  3,254 | 8.0%  (6.6 – 9.5%)  9.3%  (7.8 – 11.0%)  9.8%  (8.2 – 11.5%)  10.3%  (8.6 – 12.2%)  10.3%  (8.6 – 12.2%)  126 | 6.4%  (5.8 – 7.0%)  7.1%  (6.5 – 7.7%)  7.7%  (7.1 – 8.4%)  8.0%  (7.3 – 8.7%)  8.2%  (7.5 – 9.0%)  480 | 5.9%  (5.3 – 6.5%)  6.3%  (5.7 – 7.0%)  6.6%  (5.9 – 7.2%)  6.9%  (6.3 – 7.7%)  7.0%  (6.3 – 7.7%)  380 | 4.0%  (3.4 – 4.8%)  4.4%  (3.7 – 5.1%)  4.5%  (3.8 – 5.3%)  4.5%  (3.8 – 5.3%)  4.5%  (3.8 – 5.3%)  140 | 2.4%  (1.5 – 3.6%)  2.4%  (1.5 – 3.6%)  2.4%  (1.5 – 3.6%)  2.4%  (1.5 – 3.6%)  2.4%  (1.5 – 3.6%)  19 |
| Minor outpatient complications  1 year rate  2 years rate  3 years rate  4 years rate  5 years rate  total no. of events | 24.0%  (23.6 – 24.3%)  27.1%  (26.8 – 27.5%)  29.1%  (28.7 – 29.5%)  30.5%  (30.1 – 30.9%)  31.8%  (31.3 – 32.2%)  15,792 | 27.8%  (27.4 – 28.3%)  31.6%  (31.1 – 32.1%)  33.9%  (33.4 – 34.4%)  35.7%  (35.2 – 36.2%)  37.3%  (36.7 – 37.9%)  12,581 | 23.8%  (21.5 – 26.1%)  26.8%  (24.4 – 29.3%)  28.1%  (25.6 – 30.6%)  29.4%  (26.7 – 32.1%)  29.8%  (27.0 – 32.7%)  366 | 19.4%  (18.5 – 20.4%)  21.8%  (20.8 – 22.9%)  23.5%  (22.4 – 24.6%)  24.5%  23.3 – 25.6%)  25.2%  (24.0 – 26.5%)  1,476 | 13.8%  (13.0 – 14.7%)  15.3%  (14.3 – 16.2%)  16.3%  (15.4 – 17.4%)  16.8%  (15.8 – 17.9%)  17.2%  (16.1 – 18.3%)  923 | 10.8%  (9.8 – 11.9%)  11.8%  (10.7 – 12.9%)  12.3%  (11.1 – 13.5%)  12.4%  (11.2 – 13.6%)  12.7%  (11.4 – 14.0%)  382 | 7.7%  (6.0 – 9.7%)  8.1%  (6.4 – 10.2%)  8.1%  (6.4 – 10.2%)  8.1%  (6.4 – 10.2%)  8.1%  (6.4 – 10.2%)  64 |
| At least one secondary OAFs  1 year rate  2 years rate  3 years rate  4 years rate  5 years rate  total no. of events | 6.0%  (5.8 – 6.2%)  10.1%  (9.8 – 10.3%)  13.5%  (13.2 – 13.8%)  16.4%  (16.0 – 16.7%)  18.9%  (18.5 – 19.4%)  7,272 | 4.9%  (4.7 – 5.2%)  8.8%  (8.5 – 9.1%)  12.4%  (12.0 – 12.7%)  15.6%  (15.1 – 16.1%)  18.7%  (18.1 – 19.2%)  4,727 | 7.5%  (6.1 – 9.0%)  13.2%  (11.3 – 15.3%)  17.6%  (15.2 – 20.1%)  21.5%  (18.6 – 24.5%)  24.0%  (20.2 – 28.1%)  202 | 8.1%  (7.4 – 8.8%)  13.5%  (12.7 – 14.5%)  17.5%  (16.5 – 18.6%)  20.3%  (19.1 – 21.5%)  22.3%  (20.9 – 23.7%)  1,028 | 8.7%  (8.0 – 9.5%)  13.3%  (12.4 – 14.2%)  16.6%  (15.6 – 17.7%)  18.7%  (17.5 – 19.9%)  20.0%  (18.7 – 21.4%)  866 | 8.6%  (7.7 – 9.7%)  11.6%  (10.5 – 12.8%)  13.8%  (12.5 – 15.1%)  14.4%  (13.0 – 15.7)  14.5%  (13.1 – 15.9%)  393 | 4.7%  (3.4 – 6.4%)  6.3%  (4.7 – 8.2%)  7.0%  (5.3 – 9.1%)  7.8%  (5.9 – 10.0%)  8.6%  (6.5 – 11.1%)  56 |
| At least one secondary OAFs (incl. M80)  1 year rate  2 years rate  3 years rate  4 years rate  5 years rate  total no. of events | 14.9%  (14.6 – 15.2%)  20.1%  (19.8 – 20.5%)  24.4%  (24.0 – 24.8%)  28.2%  (27.7 – 28.7%)  31.3%  (30.7 – 31.8%)  12,642 | 13.1%  (12.8 – 13.4%)  17.9%  (17.5 – 18.3%)  21.9%  (21.5 – 22.4%)  25.7%  (25.1 – 26.2%)  28.8%  (28.1 – 29.4%)  8,265 | 20.9%  (18.8 – 23.1%)  26.5%  (24.0 – 29.0%)  32.2%  (29.2 – 35.2%)  36.1%  (32.5 – 39.6%)  40.1%  (35.1 – 45.1%)  380 | 19.9%  (18.9 – 20.9%)  27.4%  (26.2 – 28.6%)  32.7%  (31.3 – 34.2%)  36.9%  (35.2 – 38.5%)  40.7%  (38.7 – 42.8%)  1,807 | 19.0%  (18.0 – 20,1%)  25.8%  (24.5 – 27.1%)  31.6%  (30.0 – 33.3%)  36.8%  (34.8 – 38.8%)  39.4%  (37.0 – 41.8%)  1,443 | 17.3%  (16.0 – 18.8%)  22.8%  (21.1 – 24.6%)  27.6%  (25.4 – 29.9%)  30.6%  (27.9 – 33.4%)  31.2%  (28.3 – 34.2%)  639 | 12.1%  (9.8 – 14.7%)  15.8%  (12.8 – 19.1%)  18.9%  (15.0 – 23.1%)  21.6%  (16.9 – 26.8%)  24.2%  (17.6 – 31.5%)  108 |

**Additional file 5:** Stratified analyses for all primary endpoints visualized in **Figure 1**. Stratified analyses for all primary endpoints to detect differences in the associations for pre-existing level of care (LoC) depending on treatment group for different outcomes after proximal humeral fracture (PHF). For all models, an interaction term between LoC and treatment group (surgical treatment within 21 days after PHF yes/no) was added. The treatment group was considered time-dependent. Modelling was performed using multivariable proportional hazard Cox regression analysis for overall survival, major adverse events and thromboembolic events. In the case of all other endpoints, death was considered a competing risk event, and sub-distributional hazard ratios were determined via Fine and Gray models. Full results of the models are presented in **Additional file 3**.

| **LoC** | **Surgical treatment** | | | **Non-operative treatment** | | | **Comparing treatment** | | | **p_int_** |
| --- | --- | --- | --- | --- | --- | --- | --- | --- | --- | --- |
|  | **HR (LoC)** | **95%CI**  **(LoC)** | **P val**  **(LoC)** | **HR**  **(LoC)** | **95%CI**  **(LoC)** | **P val**  **(LoC)** | **HR**  **(OP)** | **95%CI**  **(OP)** | **P val**  **(OP)** |  |
|  | **Overall survival** | | | | | | | | | 0.241 |
| **no** | 1.00 | Ref. | n.a. | 1.00 | Ref. | n.a. | 0.89 | (0.84; 0.94) | <0.001 |  |
| **I** | 1.69 | (1.43; 1.98) | <0.001 | 1.76 | (1.52; 2.03) | <0.001 | 0.85 | (0.69; 1.05) | 0.126 |  |
| **II** | 2.26 | (2.09; 2.44) | <0.001 | 2.21 | (2.07; 2.36) | <0.001 | 0.90 | (0.83; 0.98) | 0.014 |  |
| **III** | 2.85 | (2.64; 3.08) | <0.001 | 2.80 | (2.62; 2.99) | <0.001 | 0.90 | (0.84; 0.97) | 0.008 |  |
| **IV** | 3.85 | (3.50; 4.25) | <0.001 | 3.91 | (3.63; 4.22) | <0.001 | 0.87 | (0.79; 0.96) | 0.005 |  |
| **V** | 4.31 | (3.60; 5.15) | <0.001 | 5.49 | (4.92; 6.21) | <0.001 | 0.70 | (0.57; 0.85) | <0.001 |  |
|  |  | **Major adverse events** | | | | | | |  | 0.139 |
| **no** | 1.00 | Ref. | n.a. | 1.00 | Ref. | n.a. | 0.73 | (0.69; 0.76) | <0.001 |  |
| **I** | 1.68 | (1.48; 1.90) | <0.001 | 1.61 | (1.43; 1.82) | <0.001 | 0.75 | (0.64; 0.89) | 0.001 |  |
| **II** | 1.87 | (1.76; 2.00) | <0.001 | 1.82 | (1.72; 1.93) | <0.001 | 0.75 | (0.70; 0.80) | <0.001 |  |
| **III** | 2.13 | (1.99; 2.29) | <0.001 | 2.13 | (2.01; 2.26) | <0.001 | 0.73 | (0.68; 0.78) | <0.001 |  |
| **IV** | 2.67 | (2.44; 2.92) | <0.001 | 2.90 | (2.71; 3.11) | <0.001 | 0.67 | (0.61; 0.73) | <0.001 |  |
| **V** | 3.02 | (2.55; 3.59) | <0.001 | 3.70 | (3.34; 4.10) | <0.001 | 0.59 | (0.49; 0.72) | <0.001 |  |
|  | **Thromboembolic events (or death)** | | | | | | | | | 0.556 |
| **no** | 1.00 | Ref. | n.a. | 1.00 | Ref. | n.a. | 0.70 | (0.67; 0.74) | <0.001 |  |
| **I** | 1.53 | (1.33; 1.77) | <0.001 | 1.65 | (1.45; 1.88) | <0.001 | 0.65 | (0.54; 0.79) | <0.001 |  |
| **II** | 1.91 | (1.78; 2.05) | <0.001 | 1.91 | (1.80; 2.03) | <0.001 | 0.70 | (0.65; 0.76) | <0.001 |  |
| **III** | 2.29 | (2.13; 2.47) | <0.001 | 2.36 | (2.22; 2.51) | <0.001 | 0.68 | (0.64; 0.74) | <0.001 |  |
| **IV** | 2.95 | (2.69; 3.24) | <0.001 | 3.17 | (2.95; 2.40) | <0.001 | 0.66 | (0.60; 0.72) | <0.001 |  |
| **V** | 3.63 | (3.05; 4.32) | <0.001 | 4.18 | (3.79; 4.64) | <0.001 | 0.62 | (0.51; 0.74) | <0.001 |  |
|  | **Minor outpatient complications** | | | | | | | | | <0.001 |
| **no** | 1.00 | Ref. | n.a. | 1.00 | Ref. | n.a. | 0.94 | (0.90; 0.97) | <0.001 |  |
| **I** | 0.86 | (0.74; 1.00) | 0.048 | 0.88 | (0.76; 1.01) | 0.079 | 0.92 | (0.74; 1.12) | 0.384 |  |
| **II** | 0.76 | (0.70; 0.83) | <0.001 | 0.79 | (0.73; 0.85) | <0.001 | 0.90 | (0.82; 1.00) | 0.058 |  |
| **III** | 0.55 | (0.49; 0.62) | <0.001 | 0.65 | (0.59; 0.71) | <0.001 | 0.80 | (0.70; 0.91) | <0.001 |  |
| **IV** | 0.40 | (0.33; 0.49) | <0.001 | 0.59 | (0.52; 0.68) | <0.001 | 0.64 | (0.51; 0.79) | <0.001 |  |
| **V** | 0.23 | (0.13; 0.39) | <0.001 | 0.43 | (0.32; 0.58) | <0.001 | 0.49 | (0.28; 0.87) | 0.015 |  |
|  | **Surgical or injured-related complications** | | | | | | | | | 0.552 |
| **no** | 1.00 | Ref. | n.a. | 1.00 | Ref. | n.a. | 1.33 | (1.24; 1.44) | <0.001 |  |
| **I** | 1.10 | (0.87; 1.38) | 0.426 | 1.07 | (0.80; 1.43) | 0.637 | 1.37 | (0.95; 1.96) | 0.089 |  |
| **II** | 0.88 | (0.76; 1.01) | 0.071 | 0.96 | (0.83; 1.11) | 0.578 | 1.23 | (1.02; 1.46) | 0.029 |  |
| **III** | 0.94 | (0.81; 1.10) | 0.470 | 0.80 | (0.67; 0.95) | 0.013 | 1.58 | (1.28; 1.94) | <0.001 |  |
| **IV** | 0.69 | (0.53; 0.88) | 0.003 | 0.62 | (0.47; 0.80) | <0.001 | 1.49 | (1.06; 2.08) | 0.020 |  |
| **V** | 0.41 | (0.22; 0.77) | 0.006 | 0.34 | (0.17; 0.65) | 0.001 | 1.62 | (0.65; 4.00) | 0.298 |  |

**Additional file 6:** Stratified analyses for surgical complications to detect differences in the associations for surgical treatment within 21 days after proximal humeral fracture (PHF) depending on preexisting level of care (LoC). Fine and Gray model were used to determine sub-distributional hazard ratios to address for the competing risk event (death). An interaction term between LoC and treatment group was added. Treatment group was considered time-dependent as well as the length of time since PHF. Hazard ratio (HR) comparing surgical with reference to non-operative treatment with 21 days after PHF were plotted. If HR >1, then surgical treatment is associated with higher risk for the related event. The detailed data can be found in the table **Additional file 7**.


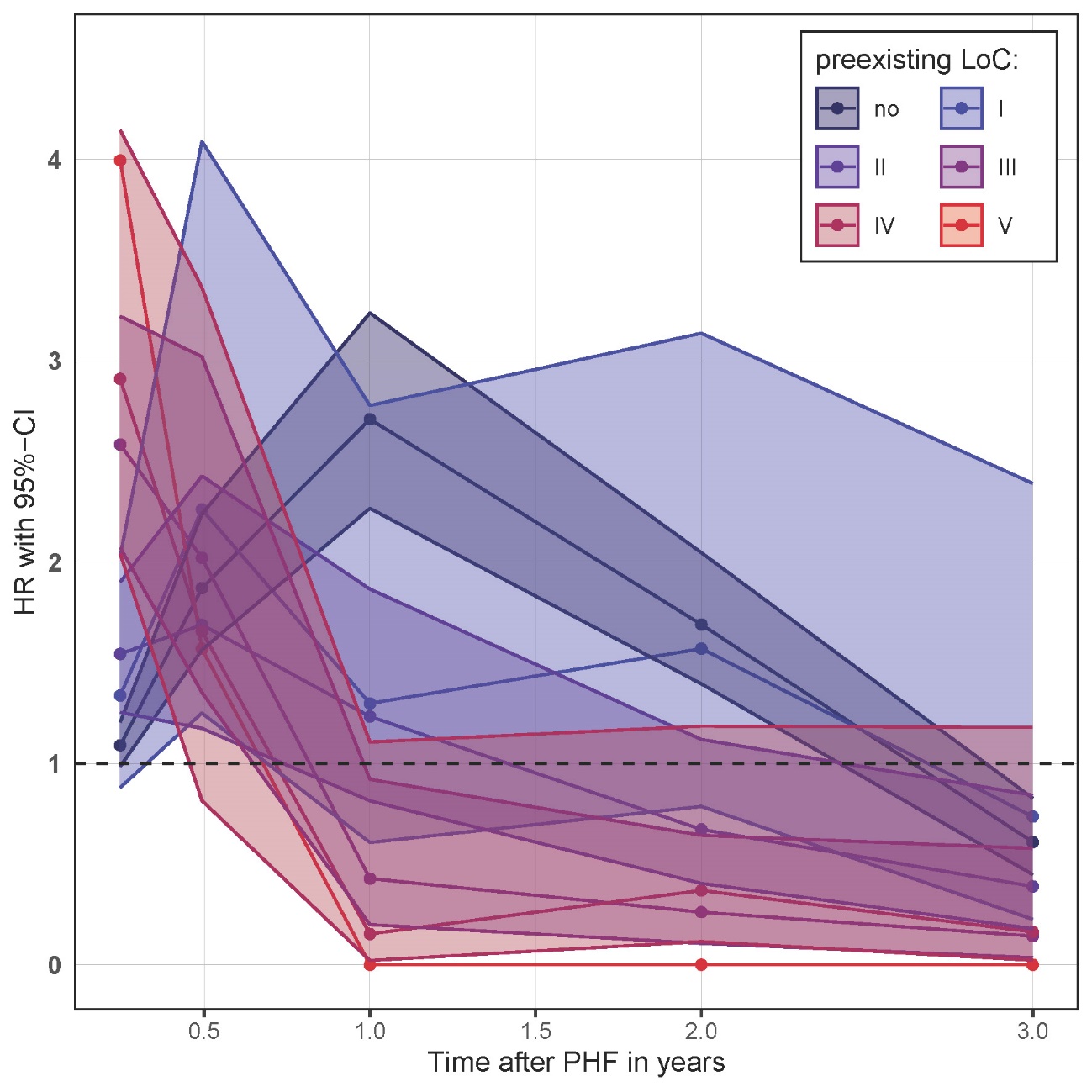


**Additional file 7**: Stratified analyses for surgical complications to detect differences in the associations for surgical treatment within 21 days after proximal humeral fracture (PHF) depending on preexisting level of care (LoC). Fine and Gray model were used to determine sub-distributional hazard ratios to address for the competing risk event (death). An interaction term between LoC and treatment group was added. Treatment group was considered time-dependent as well as the length of time since PHF. Hazard ratio (HR) comparing surgical with reference to non-operative treatment with 21 days after PHF were plotted. If HR >1, then surgical treatment is associated with higher risk for the related event. The data is visualized in **Additional File 6**.

| **Time after PHF** | **0 – 90 days** | **90 – 180 days** | **180 – 365 days** | **365 – 730 days** | **730 – 1095days** |
| --- | --- | --- | --- | --- | --- |
| **No LoC** | 1.09 (0.99 – 1.21) | 1.87 (1.56 – 2.24) | 2.71 (2.27 – 3.24) | 1.69 (1.40 – 2.05) | 0.67 (0.45 – 0.83) |
| **LoC I** | 1.34 (0.88 – 2.03) | 2.26 (1.25 – 4.09) | 1.30 (0.61 – 2.78) | 1.57 (0.79 – 3.14) | 0.74 (0.23 – 2.39) |
| **LoC II** | 1.54 (1.25 – 1.90) | 1.67 (1.17 – 2.43) | 1.23 (0.81 – 1.87) | 0.67 (0.40 – 1.12) | 0.39 (0.18 – 0.84) |
| **LoC III** | 2.58 (2.07 – 3.22) | 2.02 (1.35 – 3.02) | 0.43 (0.20 – 0.92) | 0.26 (0.11 – 0.64) | 0.14 (0.04 – 0.58) |
| **LoC IV** | 2.91 (2.04 – 4.15) | 1.66 (0.82 – 3.37) | 0.15 (0.02 – 1.11) | 0.37 (0.12 – 1.19) | 0.16 (0.02 – 1.18) |
| **LoC V** | 4.00 (1.57 – 10.2) | 1.57 (1.20 – 12.5) | n.a. |  |  |
